# Model-Driven Controlled Alteration of Nanopillar Cap Architecture Reveals its Effects on Bactericidal Activity

**DOI:** 10.3390/microorganisms8020186

**Published:** 2020-01-28

**Authors:** Taiyeb Zahir, Jiri Pesek, Sabine Franke, Jasper Van Pee, Ashish Rathore, Bart Smeets, Herman Ramon, Xiumei Xu, Maarten Fauvart, Jan Michiels

**Affiliations:** 1 Centre of Microbial and Plant Genetics, 3001 KU Leuven, Belgium; 2 Flanders Institute for Biotechnology (VIB)-KU Leuven Center of Microbiology, 3001 Leuven, Belgium; 3 Division of Mechatronics, Biostatistics and Sensors (MeBioS), 3001 KU Leuven, Belgium; 4 Interuniversity Microelectronics Centre (imec), 3001 Leuven, Belgium

**Keywords:** nanostructured surface, antibacterial surface, bacteriolysis, nanopillars

## Abstract

Nanostructured surfaces can be engineered to kill bacteria in a contact-dependent manner. The study of bacterial interactions with a nanoscale topology is thus crucial to developing antibacterial surfaces. Here, a systematic study of the effects of nanoscale topology on bactericidal activity is presented. We describe the antibacterial properties of highly ordered and uniformly arrayed cotton swab-shaped (or mushroom-shaped) nanopillars. These nanostructured surfaces show bactericidal activity against *Staphylococcus aureus* and *Pseudomonas aeruginosa*. A biophysical model of the cell envelope in contact with the surface, developed ab initio from the infinitesimal strain theory, suggests that bacterial adhesion and subsequent lysis are highly influenced by the bending rigidity of the cell envelope and the surface topography formed by the nanopillars. We used the biophysical model to analyse the influence of the nanopillar cap geometry on the bactericidal activity and made several geometrical alterations of the nanostructured surface. Measurement of the bactericidal activities of these surfaces confirms model predictions, highlights the non-trivial role of cell envelope bending rigidity, and sheds light on the effects of nanopillar cap architecture on the interactions with the bacterial envelope. More importantly, our results show that the surface nanotopology can be rationally designed to enhance the bactericidal efficiency.

## 1. Introduction

The development of nanostructured surfaces that can prevent bacterial colonization and biofilm formation is an active subject of research [1,2]. These surfaces have enormous potential for applications in healthcare and industry, promising to limit the spread of infectious diseases [3,4,5]. Different approaches have been adopted to make surfaces resistant to bacterial attachment. Biochemical approaches generally rely on coatings of substances that are toxic to bacteria [1]. Biophysical approaches include surface topographies that prevent bacterial adhesion or kill bacteria in a contact-dependent manner [6]. Ivanova et al. found that nanopillars on cicada wings can lyse bacterial cells upon contact [7]. This discovery was followed by the development of silicon [8], titanium [9], and polymer-based [10] antibacterial surfaces with similar topographies. Most of these surfaces contain the characteristic high-aspect ratio nanopillars or nanospikes. When bacteria adhere to these nanospikes, the stretching and subsequent rupture of the cell envelope suspended between the nanospikes causes cell lysis [11].

Although a variety of antibacterial surfaces have been developed using different materials, our knowledge of how geometrical variations in these nanopillars can influence the bactericidal activity remains limited. The influence of a nanoscale topology on the bactericidal efficiency has been previously investigated [12,13], but these studies focused on non-uniform surface geometries resulting from nanofabrication techniques that inherently change multiple aspects of nanostructures at once. This is not ideal for the optimization of key geometrical features, like the height, diameter, and spacing of the nanopillars, as their effects cannot be studied independently. In this work, we describe a platform of highly ordered and uniform silicon-based nanopillars for systematically studying the effects of changes in surface topography on the interaction of bacteria with surfaces. We first describe the antibacterial properties of silicon-based surfaces with cotton swab (CS)- or mushroom-shaped regularly placed nanopillars. The CS geometry is composed of a rod-shaped nanopillar with a spherical cap. These surfaces possess bactericidal activity against two common human pathogens: *Staphylococcus aureus* and *Pseudomonas aeruginosa*. We developed a biophysical model ab initio from the infinitesimal strain theory to describe the effects of the cell envelope coming in contact with nanopillar caps. In agreement with previous studies, our biophysical model also shows that the adhesion of the bacterial envelope to the CS nanopillar caps induces a tension in the cell envelope suspended between the nanopillars, which results in rupturing of the cell envelope. The model also predicts that nanopillars with flat caps (as opposed to spherical) will not induce tension high enough to cause rupturing of the bacteria envelope. This is in agreement with our experimental observations that nanopillars with flat caps lose their ability to kill the bacteria. We further use the platform of highly ordered and uniform silicon nanopillars to alter the CS nanopillar cap size without changing other aspects of the nanopillar geometry. Measurement of the bactericidal activities of these surfaces confirms another model prediction that nanopillars with bigger caps shall be more efficient in killing bacteria. These results show that nanostructured surfaces can be rationally designed for optimal bactericidal activity.

## 2. Materials and Methods

### 2.1. Silicon Nanopillar Fabrication

The highly ordered silicon-based surfaces were produced by deep ultraviolet immersion lithography and plasma etching [14,15]. Silicon dioxide and gold were deposited on the base silicon pillars by physical vapor deposition (PVD) using the Pfeiffer Spider 630 tool. Before the sputter coating, silicon surfaces were cleaned with ultraviolet light and oxygen to improve the adherence of the deposited material. To further enhance the attachment of gold caps on silicon nanopillars, the gold coated surfaces were annealed for 10 min at 120 °C.

### 2.2. Preparation of Surfaces for Experiments

To prepare the nanostructured surfaces for experiments, they were cut into small square discs of 1 cm by 1 cm by manual dicing with a diamond pen. Debris released during cutting was removed by nitrogen gas spraying. Samples were cleaned with ultraviolet light and oxygen using a Jetlight UVO-cleaner for 15 min.

### 2.3. Measurement of Bactericidal Effects of Surfaces

All experiments were performed on the following strains: *S. aureus* SH1000 [16] and *P. aeruginosa* PA14 [17]. To measure the bactericidal effects of surfaces, bacteria were grown on Luria-Bertani (LB) agar plates. The next day, stationary phase cells from colonies were suspended in PBS and the optical density of cell suspension was adjusted to 0.3 at 595 nm. After 2 h, the cell suspension was further diluted 100 or 200 times to make the initial cell suspension. In total, 800 μL of the initial cell suspension was incubated at 37 °C with the surfaces in 24-well plates (Greiner Bio-One, Kremsmünster, Austria). The plates were covered with a breathable sealing membrane (Sigma-Aldrich, St. Louis, Missouri, USA) and kept in a humidity-controlled environment during incubation. After 3 and 18 h, the wells were homogenized by pipetting up and down and 20 μL of the suspension was taken out for plating and counting. Cells were serially diluted in 25 mM MgSO_4_ solution and plated in duplicates on LB agar plates supplemented with 25 mM MgSO_4_. Bacterial survival was measured by colony-forming unit counts after overnight incubation of the plates. The viable fraction was determined from the number of viable cells in the wells with surfaces and the number of viable cells remaining in the separately incubated control suspension without any surface after the same incubation period.

### 2.4. Scanning Electron Microscopy Measurements

To prepare samples for examining the morphological changes in bacteria by SEM, 800 μL of the initial bacterial cell suspension in PBS was incubated with surfaces in 24-well plates for 18 h. After incubation, surfaces were washed with PBS three times and then incubated with 2.5% EM grade glutaraldehyde dissolved in 0.1 M sodium-cacodylate buffer for 2 h in 4 °C. This was followed by washing of the surfaces with 0.1 M cacodylate buffer, followed by 1 h incubation at room temperature with 1% osmium tetra-oxide. Samples were then dehydrated with 30%, 50%, 70%, 90%, and 100% ethanol for 10 min each at room temperature. Finally, samples were dried by putting them in hexamethyldisilazane:ethanol solutions at the ratio of 1:2, 2:1, and 1:0 (v/v) for 10 min each.

### 2.5. Fluorescence Microscopy

For fluorescence microscopy measurements, we used the BacLight bacterial viability kit (LIVE/DEAD assay) from ThermoFisher. After incubation with cell suspensions, surfaces were washed with PBS and then placed in a well containing 1 mL of water with 1 μL each of SYTO 9 and propidium iodide. After 10 min of incubation, surfaces were examined by fluorescence microscopy on a Zeiss Axio Imager.Z1. The emission of fluorescent dyes was measured at 500 nm (for SYTO 9) and 635 nm (for propidium iodide).

## 3. Results and Discussion

### 3.1. Silicon Nanopillars with a Cotton Swab Geometry Are Highly Bactericidal

**Figure 1:**
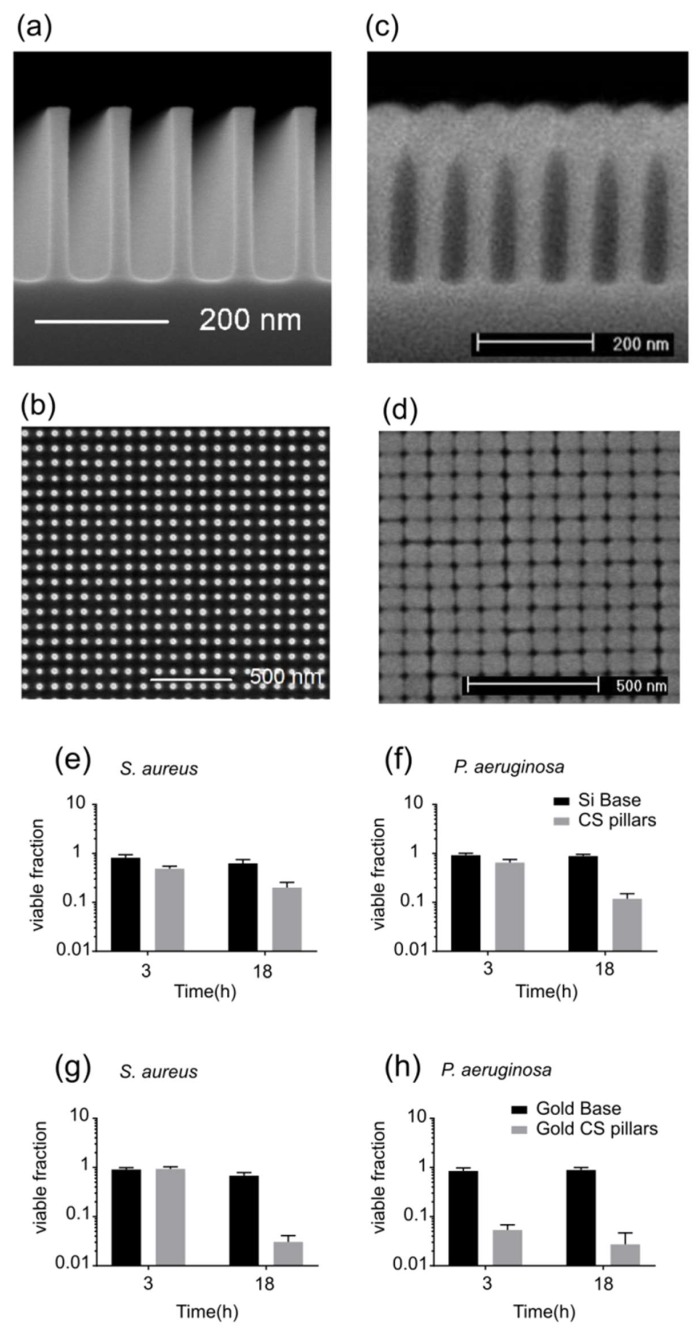
Surface topology of silicon-based cotton swab (CS) nanopillars and their bactericidal activity. (**a**–**b**) Electron micrographs show (**a**) the side-view and (**b**) the top-view of highly ordered 260 nm tall silicon nanopillars. (**c**) SEM side-view of the cotton swab (CS)-shaped nanopillars achieved by sputter coating silicon (100 nm thickness) on the nanopillars shown in (**a**) and (**b**). (**d**) SEM top-view of the CS nanopillars. (**e**–**f**) Plots show the fraction of (**e**) *Staphylococcus aureus* and (**f**) *Pseudomonas aeruginosa* cells that survived 3 and 18 h of incubation on top of the control surface (silicon base substrate) and the surface with CS nanopillars. (**g**–**h**) Plots show the fraction of (**g**) *S. aureus* and (**h**) *P. aeruginosa* cells that survived 3 and 18 h of incubation on top of the control surface (100 nm gold-coated silicon base substrate) and the surface with gold CS pillars (100 nm thickness). Viable fraction was measured by plating and colony-forming unit (CFU) counting.

The nanopillars shown in Figure 1a were fabricated on 300 mm crystalline silicon wafers (referred to as the base silicon substrate in the following text) by deep ultraviolet immersion lithography and plasma etching [14,15]. Scanning electron microscopy (SEM) investigation of the surface showed that the sizes of the pillars were uniform across the surface (Figure 1a) and the spatial arrangement of the pillars was highly ordered (Figure 1b). The pillars were 35 nm wide and square-packed with a 90 nm pitch (Figure 1a). The deposition of silicon dioxide on these nanopillars resulted in an overhang profile that looked like cotton swabs or mushrooms (Figure 1c). The size of the sphere on top of cotton swab (CS)-shaped nanopillars grew with an increasing deposition time. As spheres grew bigger, the gap between nanopillar caps became smaller. Figure 1d shows the SEM image of the surface with CS nanopillars made by depositing silicon dioxide for a duration that is equivalent to a 100 nm thick coating on a flat surface. Such a surface topography with an array of spherical caps and spaces in between can be bactericidal [11]. When bacteria adhere to a surface with such a topography, the cell envelope is non-uniformly stretched over the gaps, which in turn leads to an increase in the local tension [11]. This can cause mechanical rupturing of the cell envelope and consequently, the lysis of bacteria [18].

To assess whether the CS-shaped nanostructures possess bactericidal activity, we incubated cell suspensions of *S. aureus* and *P. aeruginosa* (see Methods for details) on top of CS pillar surfaces and base silicon substrates (1 cm^2^), respectively, in 24-well plates. The fraction of viable cells was determined by standard plate counts (see Methods for details). We also incubated the cell suspensions separately without the surfaces to account for cell death that was not due to the action of the surfaces. For CS pillars, we observed a killing efficiency of around 80% for *S. aureus* and 89% for *P. aeruginosa*, corresponding to a killing rate of 39,500 and 110,000 cells h^−1^ cm^–2^, respectively (initial counts of *S. aureus* and *P. aeruginosa* were different), over a period of 18 h (Figure 1E,F). In contrast, the base silicon substrate showed no bactericidal activity (Figure 1E,F). Previously, the bactericidal activity of silicon nanopillars was shown to be independent of the surface chemistry [8], and it was proposed that the killing of cells upon attachment to the surfaces is purely of a mechanical nature. We sputter-coated the silicon base substrate and the highly ordered silicon pillars with gold, which is well-known to be non-toxic to bacteria. For 100 nm thick depositions, the gold CS pillars were also bactericidal against both *S. aureus* and *P. aeruginosa*. The incubation of cell suspensions on top of gold CS pillars resulted in a reduction of viable cells of 97% for *S. aureus* and 98% for *P. aeruginosa*, over a period of 18 h (Figure 1G,H). This strongly suggests that the bactericidal activity of a surface with CS nanopillars is physical in nature. As a control, we also measured the bactericidal activity of base silicon substrates that were sputter-coated with gold for the same duration as the gold CS pillars. The control flat gold surfaces (without nanopillars) did not show any bactericidal activity (Figure 1G,H). This demonstrates that the CS nanopillars are essential to exerting bactericidal effects.

**Figure 2:**
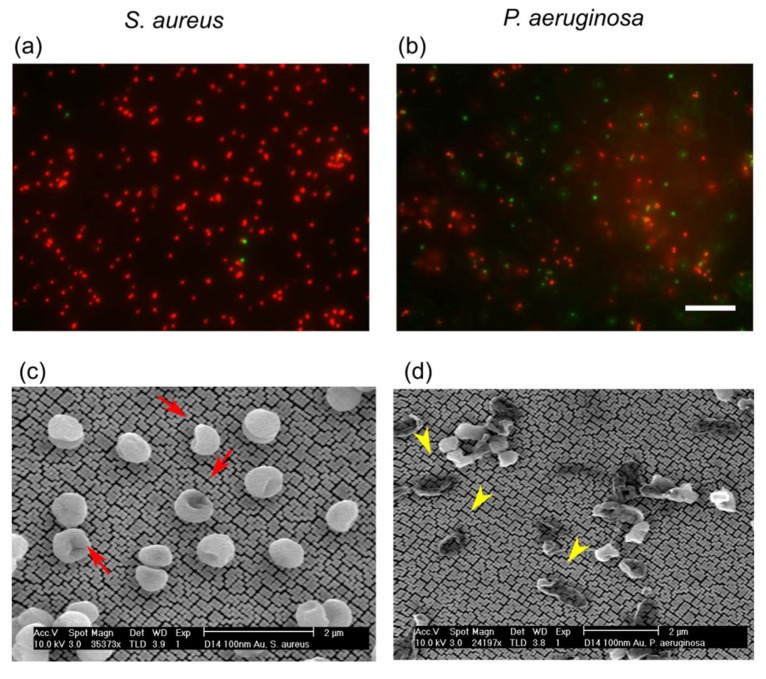
Cell lysis by cotton swab nanopillars. Micrographs from (**a**–**b**) the LIVE/DEAD assay and (**c**–**d**) SEM of *S. aureus* and *P. aeruginosa* cells incubated on 260 nm tall gold cotton swab nanopillars (100 nm deposition) for 18 h. Scale bar for fluorescence microscopy images correspond to 20 μm. Red arrows show collapsed and deformed *S. aureus* cells on the surface. Yellow arrowheads show *P. aeruginosa* cells engulfed by the nanopillars.

Cell deformation and lysis by gold CS pillars were confirmed by viability staining and SEM (Figure 2). After 18 h of incubation, the majority of *S. aureus* and *P. aeruginosa* cells attached to the surface stained red with propidium iodide, indicating a loss of structural integrity (Figure 2a,b). Fluorescence microscopy of the control surface (gold-coated silicon base substrate) showed a majority of intact, alive, and green stained cells (Figure S1). Using the viability staining assay to measure the bactericidal activity of surfaces is a popular approach; however, it does not represent the real fraction of non-viable cells in the suspension. This is because the lysed dead cells have a greater penchant to get deposited on the surface. Therefore, we did not use microscopy for the quantitative assessment of bactericidal activity and instead relied on plate counts. However, micrographs of cells are valuable to qualitatively understanding the process of lysis. For example, the patterns of cell attachment observed in the SEM micrographs were different for *S. aureus* and *P. aeruginosa*. *S. aureus* cells were deformed, but their cell envelope did not disintegrate (Figure 2c), whereas *P. aeruginosa* cells were completely engulfed by the pillars, showing that they cannot resist morphological deformations as much as *S. aureus* (Figure 2d). This is due to the difference in cell envelopes of gram-positive and gram-negative bacteria. *S. aureus*, being a gram-positive bacteria, has a much thicker cell envelope than *P. aeruginosa* (gram-negative) and is therefore more resistant to cell lysis [19]. The complete disintegration of *P. aeruginosa* cells is also highlighted in the images from the viability staining assay. In addition to red foci, patches of red smears can be seen on the surface, which indicates the engulfment of dead cells by the action of nanopillars (Figure 2b). These results are in agreement with a previous study [20] that found gram-negative bacterial species to be more vulnerable to killing by nanostructures than gram-positive species. Contrary to *P. aeruginosa* and *S. aureus*, *Escherichia coli* did not show any reduction in viable cells upon incubation on silicon CS nanopillars (Figure S2a). Fluorescence microscopy showed that *E. coli* cells did not adhere to the surface at all (Figure S2b). These results show that the CS nanopillar topology only exerts bactericidal effects upon bacterial attachment to the surface by physically rupturing the cells and inducing leakage of the cellular material.

### 3.2. Biophysical Model for Contact-Dependent Bacterial Killing by Nanostructures

To explain the bactericidal activity of nanostructured surfaces, several biophysical models have been described [11,21,22]. These models investigated the interaction of the cell envelope with a one-dimensional layout of nanopillars and provided valuable insights into the mechanism of bactericidal action. However, surface characteristics are more accurately determined by the arrangement of nanopillars in two dimensions. To enable the optimization of surface topology for maximum bactericidal activity, we decided to derive a model ab initio from the infinitesimal strain theory for 2D surfaces by incorporating a two-dimensional layout of nanopillars.

The bacterial envelope is a complex multilayered visco-elastic anisotropic medium [18,23,24]. The viscous properties of the envelope are intimately linked with bacterial growth; in other words, bacteria and consequently, the cell envelope, behave elastically in the absence of growth or on a short time scale compared to the cell doubling time [25]. For our experiments, we used stationary phase cells suspended in PBS buffer without any nutrients in order to avoid growth or biofilm formation during incubation on surfaces. The absence of growth was also confirmed experimentally from a control suspension incubated in a well without any surface. The electron micrographs shown in Figure 2 confirm the absence of biofilm formation. Additionally, Hwang et al. [24] reported linear elastic behavior of the bacterial envelope with negligible strain stiffening up to 50% of the area expansion. While it was reported that anisotropy of the rod-like bacteria’s envelope, like that of *P. aeruginosa,* is essential to explaining their observed behavior during growth and cell division [23,26], the reported anisotropy [27] is much smaller in scale than the observed variation of the overall bacterial envelope stiffness [28]. Considering all of this, in our qualitative model, which evaluates the impact of various degrees of adhesion on the tension induced in the envelope, we simplify the cell envelope to a linear elastic homogeneous isotropic medium fully characterized by its mechanical properties, while we consider the nanopillars to be rigid. Under these conditions, the infinitesimal strain theory for thin sheets is a good candidate for estimating the tension induced in the envelope. Furthermore, the elastic properties of the envelope can be expressed by a simple linear relation between the strain tensor E and tension tensor, T=kE, where k is the stiffness of the envelope. When the cell doubling time is not much larger than the adhesion maturation time or in the case of significant extracellular polysaccharide (EPS) production, for example, when the environment is abundant with nutrients, the viscous properties of the cell envelope, including dissipation of the induced stress and the dynamics of adhesion formation, have to be taken into account. This increases the complexity of the model.

The cell envelope of bacteria can be broadly classified into gram-positive and gram-negative [19] and they differ substantially in their thickness and rigidity [28]. This means that not only the stiffness, but also the bending rigidity, of the cell envelope should be considered in order to describe the mechanical properties of a cell accurately. We incorporated bending rigidity into the effective adhesion energy using the model described in [29,30] (see Supplementary Materials Section 1 for details). The mechanical interaction between the surface and the envelope is mediated by adhesion. In this model, we assumed a constant homogeneous adhesion energy density, whenever the envelope was in contact with the surface, and zero otherwise, as suggested by [11]. Although this approach neglects the intricate details of the adhesion interaction [31], we deem it sufficient for qualitatively describing the macroscopic effects of adhesion on a long timescale because the timescale of the experiment vastly exceeded the maturation time of adhesion. Extracellular polysaccharide (EPS) production can also possibly affect the adhesion strength and contact area between the bacteria and the surface, but significant EPS production on a similar surface was only observed when a substantial additional pressure to bacteria was applied [32]. These considerations justify our choice and also allow us to combine the adhesion energy density with the bending rigidity to produce a single quantity, the local effective adhesion energy $\varepsilon^{\prime }\left(x\right)$ (see Supplementary Materials Equation (1) for the exact definition).

By calculating the minimum of the free energy functional (see Supplementary Materials Sections 1 and 2), we found that the tension tensor at any point of the cell envelope T(x) is isotropic, T(x)=τ(x)I, where I is the identity tensor, and thus can be fully characterized by a scalar tension τ(x). The actual value of the scalar tension field can be further determined by the minimum of the free energy functional F,

(1) $$F=k\int^{\;}dS\left(x\right)\frac{\tau {\left(x\right)}^{2}-k\varepsilon^{\prime }\left(x\right)}{{\left[k+\tau \left(x\right)\right]}^{2}},$$

over all possible shapes of the envelope. The minimization of the free energy is constrained by the initial bacterial surface area $S_{0}$:

(2) $$S_{0}=\int^{\;}dS\left(x\right)\frac{k^{2}}{{\left[k+\tau \left(x\right)\right]}^{2}},$$

which represents conservation of the cell envelope’s mass during the process of deformation of the cell induced by adhesion. Integrations in the equations above are over the deformed surface of the bacterial envelope. The situation when τ(x)=−k corresponds to a collapsed membrane and thus represents a lower bound on the tension (see Supplementary Materials Section 2 for further discussion). Note that the solution of our model is a real physical tension of the cell’s envelope, which can be, in principle, directly measured by the means of traction force microscopy, unlike the stretching parameter introduced by Pogodin et al. [11].

**Figure 3:**
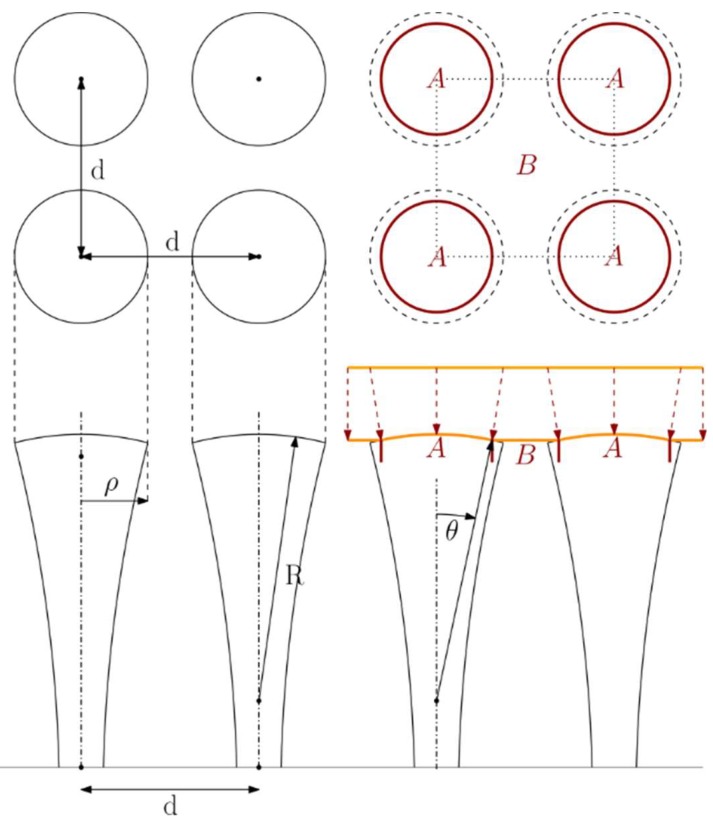
Schematic of the surface topology with modeling parameters. Figure (top) shows a representative arrangement of uniformly spaced nanopillars on a surface with a center-to-center distance d. Below is the side-view of the pillars showing the geometry of the pillar cap. R is the radius of curvature of the pillar cap, ρ is the pillar radius, θ is the wetting angle, A denotes the adhered region of the cell envelope, and B denotes the suspended region of the cell envelope. Red dashed arrows show the displacement of the cell envelope upon adherence to the pillar cap.

We approximated the morphology of nanopillars to regularly placed spherical caps with a given curvature 1/R and radius ρ (Figure 3) and estimated their parameters from SEM micrographs (Supplementary Materials Section 4). We found that this approximation describes the CS nanopillar surface quite accurately.

If we further assume that the curvature of the membrane suspended between these spherical caps can be neglected, the free energy functional (Equation (1)) can be simplified to

(3) $$F=\frac{2\pi R^{2}\left(1-cos\theta \right)\left[k{\left(\lambda -\frac{\varepsilon^{\prime }\left(R\right)}{k}\right)}^{2}-\varepsilon^{\prime }\left(R\right)\right]}{{\left[\lambda +1-\frac{\varepsilon^{\prime }\left(R\right)}{k}\right]}^{2}}+\frac{k\lambda^{2}\left(d^{2}-\pi R^{2}sin^{2}\theta \right)}{{\left[\lambda +1\right]}^{2}},$$

where θ is the wetting angle and d is the center-to-center distance between the pillars (Figure 3). $\varepsilon^{\prime }\left(R\right)$ is the effective adhesion energy for spherical caps of the radius of curvature R (see equation 8 in Supplementary Materials), and λ is the Lagrange multiplier associated with the simplified constraint (Equation (2)):

(4) $$S_{0}=\frac{2\pi R^{2}\left(1-cos\theta \right)}{{\left[\lambda +1-\frac{\varepsilon^{\prime }\left(R\right)}{k}\right]}^{2}}+\frac{d^{2}-\pi R^{2}sin^{2}\theta }{{\left[\lambda +1\right]}^{2}}.$$

Note that for the geometry depicted in Figure 3, the wetting angle θ is bounded by θ_max_ = arcsin ρ/R, as the adhesion energy needs to compensate for the huge bending energy associated with the large local curvature at the edge. This prevents the bacterial envelope from sinking along the pillar to its base. More specifically, since the area of the edge is proportional to the local radius of curvature, the contribution of bending to the free energy remains inversely proportional to the local radius of curvature and proportional to the radius of the cap. On the other hand, the bending energy of the cap is proportional to (ρ/R)^2^. By comparing these two contributions, we can estimate, using approximate values from Figure 1C, that the bending energy of the edge alone will be at least a hundred times larger than the bending energy of the cap itself. Also note that this limitation does not exist for nanopillar geometries with a smooth, locally flat transition, like those discussed by Pogodin et al. [11]. Consequently, the local tension is homogeneous in each of the respective regions. To be more specific, the local tension in the suspended region is given by τ(x)=kλ, where x∈B, and in the adhering region by $\tau \left(x\right)=k\lambda -\varepsilon^{\prime }\left(R\right)$, where x∈A. As the effective adhesion energy has to be positive for the envelope to adhere to the surface, these equations demonstrate that the local tension is typically lower in the adhering region than in the suspended region. Further analysis shows that adhesion induces compression of the cell envelope over the adhering region and expansion over the gaps (see Supplementary Materials Section 3 for more details) because the energetic gains from the adhesion overcome the cost from the associated deformation (Equation (3)). In Table 1, some suggestions regarding optimal design parameters are given. We determined that with decreasing effective adhesion or increasing stiffness of the cell envelope, the optimal radius of curvature R with respect to the pillar size ρ increases. Also note that independent of the adhesion or stiffness values, the optimal design always corresponds to tightly packed pillars, i.e., the pillar radius ρ is always half of the pitch distance d. We also provide the maximal induced tension τ with respect to the effective adhesion ε’ as a measure of the design‘s yield and fraction τ/k, which can be linked to the maximal deformation and consequently, to the bactericidal activity.

By using the geometrical properties of the surfaces like the CS nanopillar cap curvature estimated from SEM images, we numerically estimated (see Supplementary Materials Section 4) the induced tension in the cell envelope for gold CS nanopillars corresponding to 100 nm thick gold deposition on silicon nanopillars. According to our estimation, this surface induced tension in the range of 155 to 592 mN/m at the maximal cover and at the minimum of the free energy, the tension was in the range of 5 to 203 mN/m. The reported critical tension for the bacteria membrane’s rupture is between 30 and 75 mN/m [24,33], values which can be exceeded by the induced tension in the envelope for the CS nanopillars. These results are in agreement with our experimental observations and support the hypothesis that stretching of the cell envelope over the gaps leads to mechanical stress, which can cause rupturing of the bacterial membrane in the suspended region and subsequently lead to lysis. Moreover, this interpretation is also in agreement with that of Pogodin et al. [11] and it corroborates the catastrophic disintegration of the *P. aeruginosa* cell envelope and deflation of *S. aureus* (due to cytoplasm leakage) by CS-shaped nanopillars (Figure 2).

In the future, bactericidal surface topologies need to be translated to materials that are amenable to scalable production. Polymers like Polydimethylsiloxane (PDMS) and polymethyl methacrylate (PMMA) are amenable to scalable production and can be used to imprint bactericidal topologies for various purposes, like the prevention of bacterial colonization on implants and in hospitals, to limit the transmission of pathogens. Although the model presented here can be applied to study the interaction of microbes with different topologies and nanostructured surfaces made of different materials, some changes may be necessary. In this model, we assumed that nanopillars are rigid, which may not hold true for other materials. The deformation of flexible nanopillars can reduce their bactericidal activity, as it would allow relaxation of the built-up stress in the cell envelope. Adhesion of the microbes on the surface is another important factor to consider. Without sufficiently large adhesion, the CS nanopillars cannot kill bacteria. This is a beneficial trait for applications such as implants, where we selectively want to kill the microbes that adhere and form a biofilm, whereas this trait is not desirable for bactericidal surfaces that are meant to prevent the transmission of pathogens.

### 3.3. Pillar Cap Geometry Influences the Bactericidal Activity

**Figure 4:**
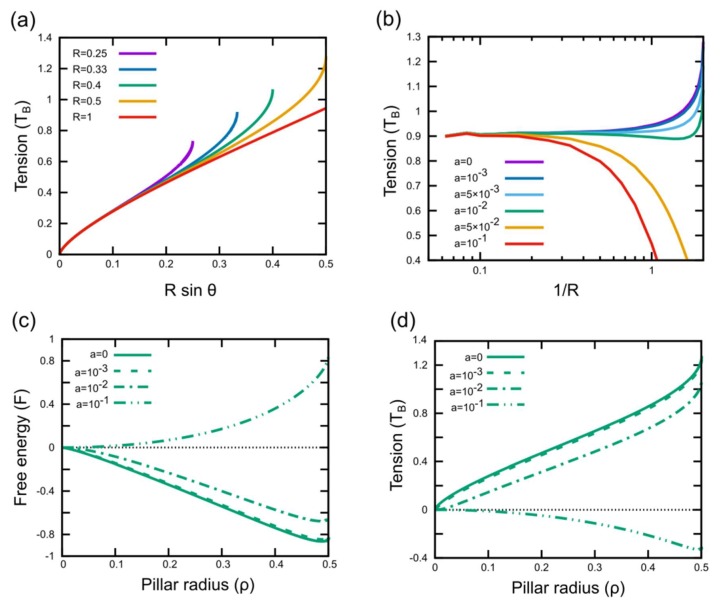
Dependency of tension in the suspended region of the cell envelope and the free energy on surface topography and adhesion. (**a**) Plot shows the monotonic increase of tension in the suspended region with an increase in the wetting angle θ. Different lines correspond to various radius values of curvatures of the pillar cap for a cell envelope without any bending rigidity. (**b**) Plot shows how tension in the suspended region changes with the pillar cap curvature. Different lines correspond to various levels of bending rigidity of the cell envelope. (**c**–**d**) Plots show how free energy (**c**) and tension in the suspended region (**d**) change with the pillar radius. Different line trends correspond to different levels of bending rigidity (shown as a). The dotted line at y = 0 in (**c**–**d**) represents the situation when there is no change in free energy or tension, respectively, with respect to the initial configuration of the cell envelope. For a sufficiently high bending rigidity (a = 0.1), the change in free energy is positive, indicating that the adhesion of cells to the surface is energetically unfavorable.

**Figure 5:**
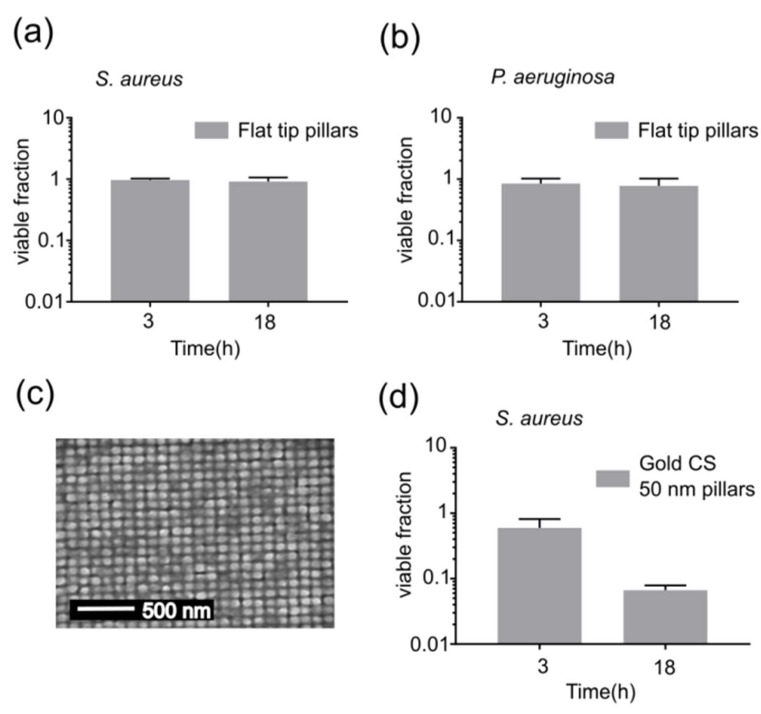
Effects of surface topology on the bactericidal activity. (**a**–**b**) show the fraction of (**a**) *S. aureus* and (**b**) *P. aeruginosa* cells that survived 3 and 18 h of incubation on top of 260 nm tall flat-tip silicon nanopillars. (**c**) SEM top-view of 50 nm tall ordered silicon nanopillars sputtered with gold (100 nm thickness). (**d**) shows the fraction of *S. aureus* cells that survived 3 and 18 h of incubation on top of short gold cotton swab (CS) nanopillars shown in (**c**).

We further used the biophysical model described above to investigate the effects of changes in pillar cap geometry on the bactericidal activity. Since the cells lyse due to rupturing in the suspended region of the cell envelope, bactericidal activity can be assumed to be proportional to tension in the suspended region. The model suggests that the change in the total area of the cell envelope induced by the curvature of pillars is essential for bactericidal activity (Figure S12). As the wetting angle increases, the change in the total area becomes larger and more tension is induced in the suspended envelope (Figure 4a). Besides the wetting angle, the tension induced in the cell envelope by nanopillars is also affected by the curvature of the pillar cap and bending rigidity of the cell envelope. The effect of bending rigidity is more pronounced in the case of nanopillar caps with large curvatures (Figure 4b). For a given pillar radius (or width), an increase in bending rigidity leads to the poor adhesion of cells on pillars (Figure 4c) and therefore, causes less tension in the suspended region (Figure 4d). An increase in cap curvature effectively increases the available area to which cells can adhere, but also makes it harder for cells to adhere due to bending rigidity (Figure 4b). For cell envelopes with a very low bending rigidity, contact with a pillar with increased cap curvature leads to a dramatic increase in tension in the suspended region, whereas for a high bending rigidity, an increase in cap curvature leads to poor adhesion and a decrease in tension (Figure 4b). These results indicate that nanopillars with a flat cap, like the nanopillars shown in Figure 1a (having, in extreme cases, an infinite curvature at the sharp edges), should be much less effective than the CS-shaped nanopillars in the same experimental condition due to the poor adhesion of cells. This is further supported by our numerical results, which estimate the induced tension in the suspended region to be between 0.12 and 16 nN/m (Supplementary Materials Section 4); much below the tension needed to cause a rupture of the cell envelope. Even for equally-sized (as compared to the area of 100 nm CS nanopillar caps) flat caps, the tension is estimated to be much lower than that of spherical caps of CS nanopillars; 0.7–75 nN/m. The prediction that flat capped nanopillars will be less effective was confirmed by experiments. Both *S. aureus* and *P. aeruginosa* did not experience any measurable loss of viability when incubated on the flat-tip pillars for 18 h (Figure 5a,b).

**Figure 6:**
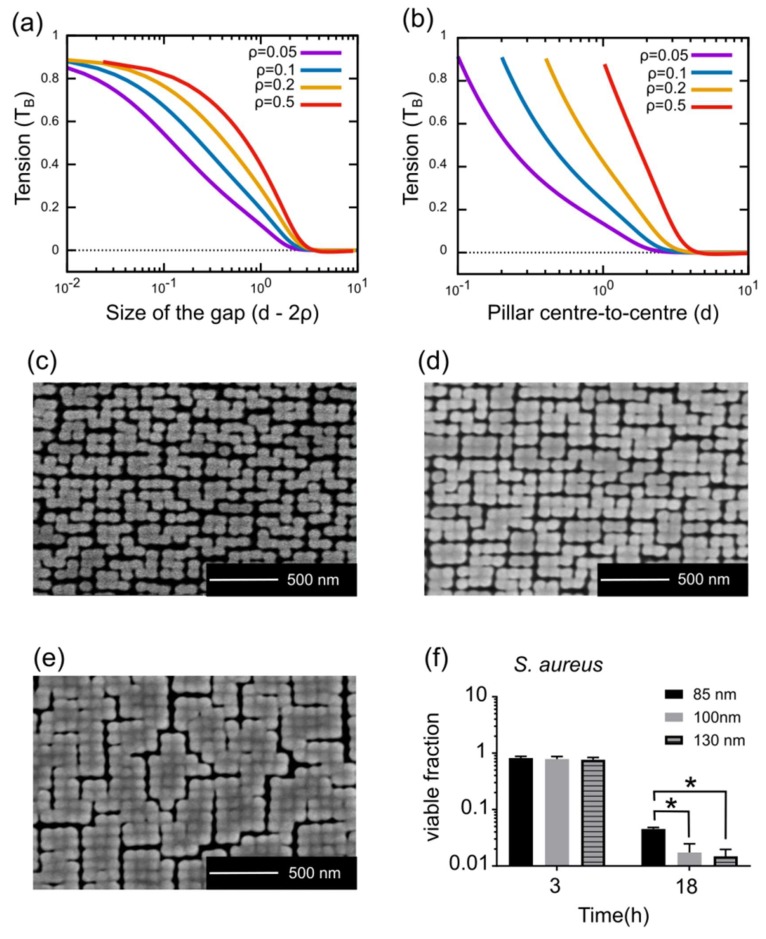
The effects of pillar cap size on bactericidal activity. (**a**–**b**) shows the dependency of tension in the suspended region on the size of the gap between (**a**) pillar caps and (**b**) the center-to-center pillar distance. Different lines correspond to various radii of the pillars. (**c**–**e**) shows SEM top-views of surfaces made by different durations of gold sputtering on 260 nm tall silicon nanopillars. The duration is equivalent to sputtering a (**c**) 85, (**d**) 100, and (**e**) 130 nm thick coating on a flat surface. (**f**) shows the fraction of *S. aureus* cells suspended in water that survived 3 and 18 h of incubation on top of the cotton swab-shaped nanopillars with a gold cap shown in A, B, and C. * indicates P-value < 0.005, measured by an unpaired, two-tailed parametric t-test.

The biophysical model also implies that the spherical caps are sufficient for killing the cells, even without the pillars, and that the height of the pillars should not have a major influence on the bactericidal efficiency. To make a surface with only spherical caps separated by gaps in between, we deposited gold on short (50 nm in height) silicon pillars. When the pillars were only 50 nm high, the gold deposition totally engulfed the pillars. SEM investigation showed a similar surface topography to the surface with 260 nm tall CS pillars (Figure 5c). This surface was also bactericidal towards *S. aureus*, killing around 60% of the cells in 18 h (Figure 5d). In the model, we assumed that the nanopillars were rigid. Nevertheless, we observed a clustering of the nanopillars to larger domains after the deposition of gold, with a typical pitch distance of 86 nm (see Figure 6c–e), indicating that the nanopillars were not perfectly rigid. We did not observe the clustering of nanopillars to larger domains in the case of 50 nm tall nanopillars (Figure 5c), as they have a much higher bending rigidity. While the lower bending rigidity of the 260 nm tall nanopillars can relax some of the strain induced in the cell envelope, and thus inhibit the bactericidal activity, clustering of the caps locally increases the pillar density and thus effectively decreases (by 5%) the spacing between the pillar caps which, as we will discuss later, enhances the bactericidal activity. This may explain why the tall 260 nm CS pillars have a higher efficiency than the short 50 nm CS pillars. One of the common problems with cleaning nanostructured surfaces is the capillary-induced clustering of nanostructures. The CS profile shows bactericidal activity due to the caps and not the underlying nanopillars. Therefore, there is no need to make the slender high aspect ratio needles described in previous literature, for which we would expect a lot of damage after cleaning. The observation that caps without pillars are also bactericidal is very promising since the use of mechanically robust structures would enable real applications.

Next, we used the biophysical model to determine the effects of the size of the nanopillar caps and spacing between them on the efficacy of bacterial killing. For a given pillar radius (or width), an increase in size of the gap leads to a decrease in the envelope tension (Figure 6a,b). Increasing the size of caps of CS nanopillars decreases the gap size and also provides more area for adhesion. Therefore, CS nanopillars with bigger caps should possess higher bactericidal activity. Precise calculations yielded a value of surface-induced tension in the range of 115 to 456 mN/m and 4 to 186 mN/m for 85 nm thick gold deposition (as opposed to 155 to 592 mN/m and 5 to 203 mN/m for 100 nm thick gold deposition) at the maximal cover and minimum of free energy, respectively. To confirm the prediction that bigger caps are more efficient, we made three distinct geometries of CS nanopillars. By modulating the duration of gold sputter coating, we achieved gold CS pillars with different sizes of spherical caps (Figure 6c–e). The different durations of coating corresponded to the deposition of a 85, 100, and 130 nm thick gold coating. We tested the bactericidal activity of these surfaces against *S. aureus*. After 3 h of incubation, none of them showed any significant bactericidal activity, but after 18 h, all three surfaces eliminated more than 50% of the cells in the suspension (Figure 6f). More importantly, we observed that increasing the size of the cap increases the killing efficiency (Figure 6f), which is in agreement with the predictions of the model, which estimates that the surface 100 nm thick gold deposition increases the induced tension in the envelope by 7% to 55% with respect to 85 nm thick gold deposition, depending on the exact conditions. Together, these results demonstrate that the interaction of bacteria with nanostructured surfaces is greatly influenced by the geometry and spacing of the nanopillar caps.

Finally, we estimated the radius of curvature maximizing the induced tension at the minimum free energy for equally large pillar caps and found the optimal radius of curvature to pillar caps radius ratio in the range of 1.08–1.25 (Supplementary Materials Section 4), which are values quite close to those of hemispherical caps and surfaces described in this study.

## 4. Conclusions

In this study, we have shown that highly ordered nanopillars with spherical caps are bactericidal. Using *S. aureus* and *P. aeruginosa* as model bacteria, we showed that these CS-shaped nanopillars exert bactericidal activity towards both gram-positive and gram-negative pathogens. The bactericidal effect of CS nanopillars was demonstrated by standard plate counting, SEM imaging, and a fluorescence-based LIVE/DEAD cell viability assay. We propose a simple biophysical model for deformation of the bacterial cell envelope, which we developed ab initio from the infinitesimal strain theory. The model allowed us to qualitatively predict the bactericidal activities of various surface topographies. These predictions were confirmed by further experiments on different nanopillar geometries. Both the biophysical model and experimental evidence show that the architecture of the nanopillar caps affects the efficiency of bacterial killing. The CS nanopillars with bigger caps were more efficient in killing as they provided more surface area for the adhesion of cell envelopes, resulting in more stretching of the cell envelope that is suspended between the pillars. Our findings present a platform for the rational design of antibacterial surfaces. The described CS nanostructures are amenable to several kinds of structural alterations. For example, the pillar cap geometry can be changed by different durations of plasma etching and sputter coating. Studies with a systematic and controlled alteration of surface topography will potentially unravel crucial knowledge about bacterial interactions with nanostructured surfaces and facilitate the development of smartly designed antibacterial surfaces.

## Figures and Tables

**Table 1:** The table depicts an optimal design for various degrees of effective adhesion ε’ with respect to the cell envelope stiffness k, based on a numerical evaluation of the model (see Supplementary Materials Section 4).

ε’/k	R/ρ	ρ/d	τ/ε’	τ/k
10^0^	1.04	0.5	1.1	1.1
10^−1^	1.18	0.5	2.1	0.2
10^−2^	1.70	0.5	4.9	4.9 × 10^−2^
10^−3^	2.78	0.5	15	1.5 × 10^−2^
10^−4^	4.78	0.5	45	4.5 × 10^−3^
10^−5^	8.46	0.5	141	1.4 × 10^−3^

## Supplementary Materials

Supplementary materials can be found at https://www.mdpi.com/2076-2607/8/2/186/s1.
