# Supplementary material for: Model-Driven Controlled Alteration of Nanopillar Cap Architecture Reveals its Effects on Bactericidal Activity
Source: Microorganisms. 2020 Jan 28;8(2):186. doi: 10.3390/microorganisms8020186 (PMC7074768; doi:10.3390/microorganisms8020186)
Supplement: Supplementary file 1 [file microorganisms-08-00186-s001.pdf]

Supplementary information: Model-driven controlled alteration of nanopillar cap architecture reveals its effects on bactericidal activity

Taiyeb Zahir, Jiří Pešek, Sabine Franke,
Jasper Van Pee, Ashish Rathore, Bart Smeets, Herman Ramon,
Xiumei Xu, Maarten Fauvart and Jan Michiels

January 27, 2020

1 Model introduction

The starting point of our model is the infinitesimal theory for 2D membranes [3]. Within this framework, the deformation of bacteria's envelope is fully characterized by the displacement vector field $\boldsymbol{u}(\boldsymbol{x})$, which relates the initial position of any point of the envelope $\boldsymbol{x}$ to its position after the deformation $\boldsymbol{y}(\boldsymbol{x})$, i.e. $\boldsymbol{u}(\boldsymbol{x}) = \boldsymbol{y}(\boldsymbol{x}) - \boldsymbol{x}$. This vector field does not contain only deformations, but also translations and rotations. In order to separate the deformations from the global translations and rotations, the gradient of the displacement field is introduced, $U = \boldsymbol{\nabla_x u}(\boldsymbol{x})$. In the infinitesimal strain theory, we assume that all deformations are so small, that we can linearise all relevant expression. Thus, Cauchy's strain tensor is simplified to

$$E = \frac{1}{2}\left[U + U^T\right],$$

where U^T denotes transpose of the displacement gradient U.

In order to simplify the analytical investigation of the problem at hand, we further assume that the bacteria envelope is a homogeneous isotropic elastic medium, thus the tension in the membrane represented by the tension tensor T is related to the strain tensor E simply through an elastic constant k, $T = kE$. As there are large structural differences between gram-negative and gram-positive bacteria, the bending rigidity also needs to be taken into account. Here, we use the model developed by Helfrich for a lipid bilayer [9, 11]. Finally, the work of the adhesion, which drives the deformation of the envelope in contact with a surface, is represented in our model by the adhesion energy density $\varepsilon(\boldsymbol{x})$. Here, we use a constant adhesion energy density whenever a envelope is in contact with the surface and zero otherwise, as suggested by [22], rather than applying more advance models discussed in [14, 15] in order to reduce the complexity of the model to its essentials. This altogether leads to the free energy of the envelope adhering to a surface in the form of

$$\mathcal{F} = \int \mathrm{d}S_0(\boldsymbol{x}) \left[\frac{1}{8}k \operatorname{Tr} E^2 - \varepsilon(\boldsymbol{x}) + a\left(\kappa_1(\boldsymbol{x}) + \kappa_2(\boldsymbol{x}) - 2\kappa_0(\boldsymbol{x})\right)^2 + a'\kappa_1(\boldsymbol{x})\kappa_2(\boldsymbol{x})\right],$$

where the first term represents the elastic energy density, the second term is the adhesion energy density and the last two terms are contributions from the bending energies where a and a' are the

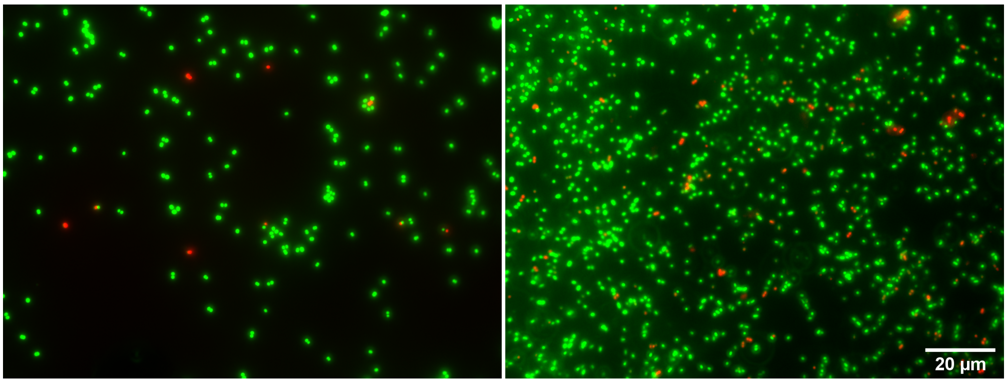

Figure 1: Micrographs show S. aureus (left) and P. aeruginosa (right) cells incubated for 18 hours on flat silicon base substrates coated with 100 nm thick gold layer and then stained with Baclight LIVE/DEAD stains.

bending rigidities and $\kappa_{1,2}(\boldsymbol{x})$ are the principal curvatures, while $\kappa_0(\boldsymbol{x})$ is the preferred curvature of the envelope.

Note, that the integration is over the original unperturbed surface S_0 associated with coordinate system $\boldsymbol{x}$, rather than over the full deformed surface S associated with the coordinate system $\boldsymbol{y}$. This reflects two important aspects of the model: First, we assume that the total number of adherent junctions is preserved during the deformation. Second, only in the coordinate system associated with the unperturbed surface the elastic and bending contributions decouple. If the free energy would be integrated over the deformed surface area of the envelope while having the same functional dependency, it would be impossible to distinguish between the situation where the area increases due to the deformation and the situation where the area increases due to the bacteria metabolism.

Also note that we consider the surface, to which the envelope adheres, as rigid. As such, it contributes to the free energy only through the adhesion energy.

Influence of the bending rigidity The bending rigidity contributes to the free energy in the same manner as the adhesion but with opposite sign. Hence, we can introduce an effective adhesion energy

$$\varepsilon'(\boldsymbol{y}) = \varepsilon(\boldsymbol{y}) - a\left(\kappa_1(\boldsymbol{y}) + \kappa_2(\boldsymbol{y}) - 2\kappa_0(\boldsymbol{y})\right)^2 - a'\kappa_1(\boldsymbol{y})\kappa_2(\boldsymbol{y}), \tag{1}$$

which is non-positive in non-adhered regions. It also implies that if the curvature of the envelope becomes too high, the envelope becomes locally nonadhesive. Thus, surfaces with high local curvature, or roughness, will exhibit *antibiofouling* behavior. This was also demonstrated on *Staphylococcus aureus* in [7], where the bacteria managed to adhere only in convex pockets of the presented structure, where the bending energy of the bacteria's envelope is minimized. It was also observed in our setup in case of *Escherichia coli*, weakly adhesive bacteria under normal conditions [19], which failed to adhere to the otherwise highly bactericidal gold coated nanopillars, see figure 2.

Free energy in the deformed configuration We typically know the deformed configuration and some quantities likes curvatures are also easier to evaluate in the deformed configuration.

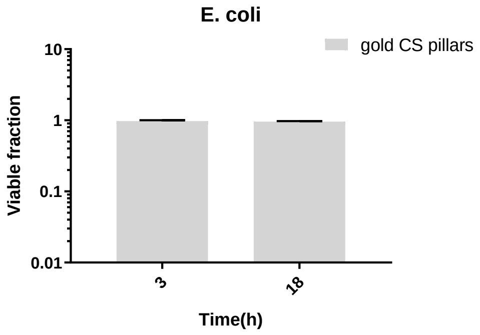

(a) Plot shows that almost all *E. coli* cells survived 3 and 18 hours of incubation period on top of nanopillars with gold deposition thickness of 100 nm.

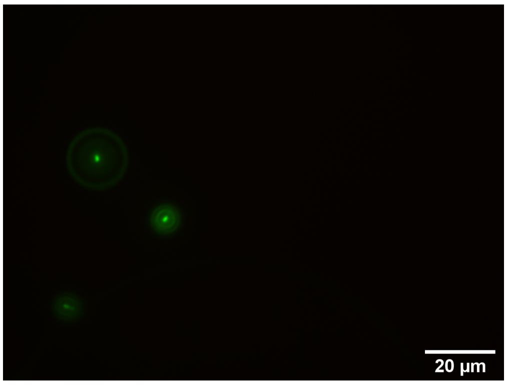

(b) The micrograph of *E. coli* cells incubated for 18 hours on top of nanopillars with gold deposition thickness of 100 nm and then stained with Baclight LIVE/DEAD stains.

Figure 2: Experimental verification that the manufactured surface is not bactericidal for weakly adhesive bacteria like *E. coli* as they practically do not interact with the surface.

Consequently, we transform the free energy into the deformed coordinate system

$$\mathcal{F} = \int \mathrm{d}S\,(\boldsymbol{y})\; \frac{\frac{1}{8}k\,\mathrm{Tr}\left(U + U^T\right)^2 - \varepsilon'(\boldsymbol{y})}{|\det\left(\mathbb{I} + U\right)|}, \tag{2}$$

where $|\det\left(\mathbb{I} + U\right)|$ is the Jacobian of the given transformation and the bending energy was incorporated to the effective adhesion energy ε'. Note that in this functional form most of the information about the original configuration is lost. In order to provide a link with the original configuration we thus have to introduce a constraint

$$S_0 = \int \mathrm{d}S(\boldsymbol{y})\; \frac{1}{|\det\left(\mathbb{I} + U\right)|},$$

which can be interpreted as the mass conservation during the envelope deformation.

2 Partial solution

It can be shown, that for a given envelope geometry, the minimum of the free energy (2) is attained for isotropic deformation

$$U = \frac{\tau(\boldsymbol{y})}{k}\,\mathbb{I}.$$

This solution also simplifies the tension tensor to

$$T = \tau(\boldsymbol{y})\,\mathbb{I},$$

so we can identify the $\tau(\boldsymbol{y})$ as the local tension, which is further determined by a minimum of the reduced free energy under the constraint

$$\mathcal{F} = k\int \mathrm{d}S\,(\boldsymbol{y})\; \frac{\tau^2(\boldsymbol{y}) - k\varepsilon'(\boldsymbol{y})}{\left[k + \tau(\boldsymbol{y})\right]^2}, \qquad S_0 = \int \mathrm{d}S\,(\boldsymbol{y})\; \frac{k^2}{\left[k + \tau(\boldsymbol{y})\right]^2}.$$

Note that, the case when $\tau(\boldsymbol{y}) = -k$ corresponds to collapsed envelope because $\det(\mathbb{I} + U) = 0$. Thus, if we start from the envelope without any external stress applied, i.e. $\tau(\boldsymbol{y}) = 0$, and assume continuous deformation of the bacteria envelope towards the non-degenerate state with the minimal free energy, it is safe to assume

$$\forall \boldsymbol{y}: \quad \tau(\boldsymbol{y}) > -k.$$

Note also, that this bound is a direct consequence of the assumption of the linear elasticity of the envelope's material, which has to be abolished in case of strong adhesion, $\tau(\boldsymbol{y}) \approx -k$.

Furthermore, it can be shown that the minimum of reduced free energy is achieved for the tension in the form

$$\tau(\boldsymbol{y}) = k\lambda - \varepsilon'(\boldsymbol{y}), \tag{3}$$

where λ is the associated (scaled) constant Lagrange multiplier, fully determined by the constraint

$$S_0 = \int \mathrm{d}S\,(\boldsymbol{y})\ \frac{k^2}{\left[k(1+\lambda) - \varepsilon'(\boldsymbol{y})\right]^2}. \tag{4}$$

This result means that the local tension $\tau(\boldsymbol{y})$ is not homogeneous over the surface, but depends locally on the geometry via the effective adhesion energy density ε'. It also means that the adhered region is under lower tension than the non-adhered region, thus the suspended region of the cell's envelope is the most vulnerable.

Note, that the lower bound on the tension translates to the lower bound for the Lagrange multiplier

$$\forall \boldsymbol{y}: \quad k(\lambda + 1) > \varepsilon'(\boldsymbol{y}). \tag{5}$$

This also means that sufficiently large adhesion $\varepsilon'(\boldsymbol{y}) > k$ leads to immediate collapse of the membrane, as in the initial unperturbed configuration $S = S_0$ and thus $\lambda = 0$.

The free energy for a given shape of the envelope is given by

$$\mathcal{F} = k \int \mathrm{d}S\,(\boldsymbol{y})\ \frac{k^2\lambda^2 - (2\lambda+1)k\varepsilon'(\boldsymbol{y}) + \varepsilon'(\boldsymbol{y})^2}{\left[k(1+\lambda) - \varepsilon'(\boldsymbol{y})\right]^2}, \tag{6}$$

where the Lagrange multiplier is given by (4).

Our model differs in several key aspects to the model introduced by Pogodin *et al.* [22]. The local stretching introduced by Pogodin *et al.* is replaced here by the actual local tension in the cell envelope and also the bending rigidity of the cell envelope is explicitly included in this model. This allow us to accurately capture the mechanical properties of both gram positive and negative bacteria and also opens the possibly to relate the model predictions directly to other experimental setups like traction force microscopy.

2.1 Simple topography

The first case we consider is a patch of the bacterial envelope completely adhering to a smooth surface. In this simple case, the effective adhesion energy density is uniform over the surface, $\varepsilon'(\boldsymbol{y}) \equiv \varepsilon'$, which allow us to find an analytic solution $\lambda = \sqrt{\frac{S}{S_0}} - 1 + \frac{\varepsilon'}{k}$, where S is the area of the patch after adhesion and S_0 is before adhesion. Note, that the tension is uniformly characterized by the ratio between the surface areas regardless of the adhesion energy density

$$\tau = k\left[\sqrt{\frac{S}{S_0}} - 1\right].$$

The associated free energy is given by

$$\mathcal{F} = S_0 \left[k \left(\sqrt{\frac{S}{S_0}} - 1 \right)^2 - \varepsilon' \right],$$

which is minimal for $S \equiv S_0$ with $\mathcal{F}_{\min} = -\varepsilon' S_0$. This also means that the patch will not adhere to the smooth surface if the effective adhesion energy density is negative, $\varepsilon' < 0$, for example due to the preferred curvature of the membrane.

This also shows that in the equilibrium there is no tension induced on the patch due to the adhesion as $\tau_{\min} = 0$. In other words there is no difference in the tension, and consequently no bactericidal activity, whether or not the patch adheres to a smooth surface, e.g. to the substrate. We confirmed this prediction with LIVE/DEAD stainig, see fig. 1 This first result already highlights the importance of the surface's topography with respect to its bactericidal activity.

Moreover, we also notice that in case the patch of the bacterial envelope is further constrained or stretched, it will adhere only if the area and the tension are below a threshold given by the effective adhesion energy,

$$S < S_0 \left[1 + \sqrt{\frac{\varepsilon'}{k}} \right]^2 \qquad \text{and} \qquad \tau < \sqrt{k\varepsilon'}.$$

3 Nanopillars with spherical caps

We have demonstrated that smooth surfaces have no bactericidal activity. To demonstrate how exactly a surface with topographical features promotes bactericidal activity, we will study a surface formed by cylindrical nanopillars regularly placed in square grid atop a substrate. Nanopillars are described by their height, their cap's radius ρ and their center-to-center distance d. Tips of the nanopillars are formed by spherical caps with curvature radius R, as depicted in the figure 3, while the portion of the bacteria envelope in contact with the surface is, due to the symmetry of the problem, characterized simply by the wetting angle θ. This provides us with a rich set of parameters influencing the tension, and consequently the bactericidal activity. Note, that for $R > \rho$ the sharp edges of the nanopillar's caps create an effective barrier preventing further adhesion towards the base of the pillars due to their high local curvature, hence the height of the pillars does not play any role in the model.

In order to simplify the analysis we will assume that the preferred curvature of the bacteria's envelope is zero, $\kappa_0 = 0$. And also that the patch of the envelope suspended between the four pillar caps, see fig. 3, is representative of the overall envelope, which is in contact with the surface. We further assume that the effect of the curvature of the envelope in the suspended region close to the pillar edges can be neglected. Inserting this geometry into the equation for the free energy (6) and constraint (4), we obtain

$$\mathcal{F} = k \frac{\left[k^2\lambda^2 - (2\lambda+1)k\varepsilon'(R) + \varepsilon'(R)^2\right]\left[2\pi R^2 (1-\cos\theta)\right]}{\left[k(\lambda+1) - \varepsilon'(R)\right]^2} + \frac{k\lambda^2\left[d^2 - \pi R^2 \sin^2\theta\right]}{(\lambda+1)^2}, \tag{7}$$

where the effective adhesion energy density is given by

$$\varepsilon'(R) = \varepsilon - \frac{4a + a'}{R^2}, \tag{8}$$

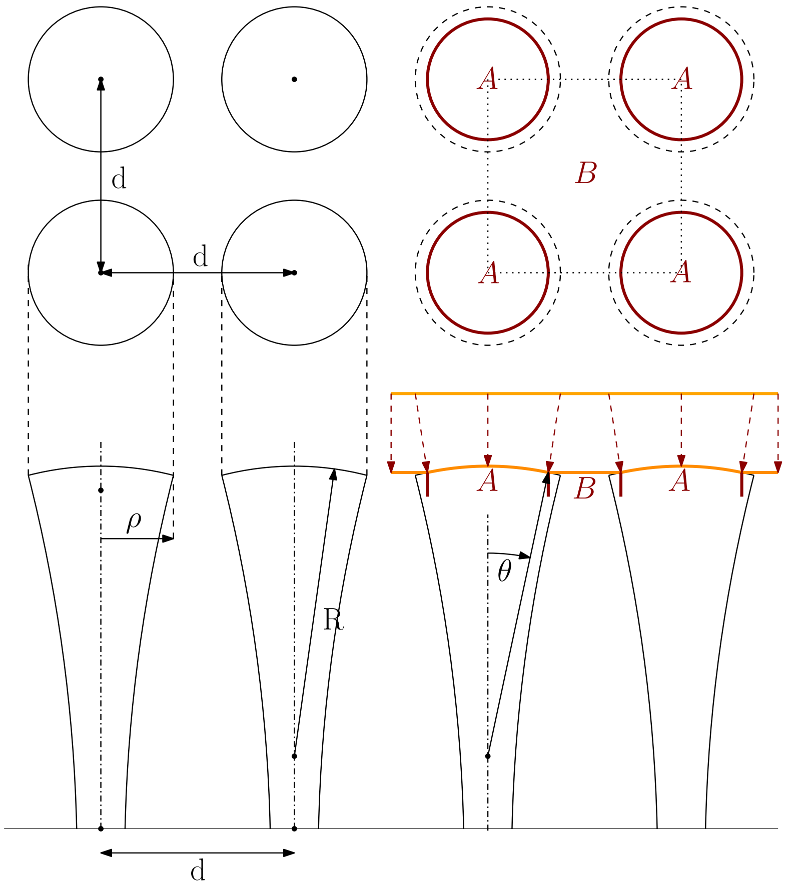

Figure 3: The geometrical representation of a general surface topography. The top view is shown above of the cross-section. The pillar caps have radius ρ, with the radius of curvature R. They are spaced in a regular square grid at a center-to-center distance d. A denotes an adhered region in the form of spherical cap with wetting angle θ and B denotes the region where the envelope is suspended between the pillars. By orange color we denote the surface of the membrane and red arrows denote the displacement field connecting identical points of the envelope before and after adhesion.

the Lagrange multiplier λ is determined by

$$g(\lambda,\theta) = \frac{k^2\left[2\pi R^2\left(1-\cos\theta\right)\right]}{\left[k\left(\lambda+1\right)-\varepsilon'(R)\right]^2} + \frac{d^2-\pi R^2\sin^2\theta}{\left(\lambda+1\right)^2} - d^2 \equiv 0 \tag{9}$$

and by the wetting angle θ obtained by further minimization of the free energy over the available surface, $\sin\theta_{\max} = \rho/R$. Note, that as the Lagrange multiplier λ implicitly depends on the angle θ, see (9), finding the minimum of the free energy with respect to the wetting angle is highly non-trivial as it effectively leads to the solution of

$$\begin{aligned} 0 &= \partial_\theta \mathcal{F}\left(\lambda,\theta\right) - \frac{\partial_\theta g(\lambda,\theta)}{\partial_\lambda g(\lambda,\theta)}\,\partial_\lambda \mathcal{F}\left(\lambda,\theta\right), \qquad \text{for} \qquad 0 \le \theta \le \theta_{\max} \\ 0 &= g(\lambda,\theta), \end{aligned} \tag{10}$$

and has to be performed mostly numerically.

The tension is then given by (3),

$$\tau(\boldsymbol{y}) = \begin{cases} k\lambda - \varepsilon + \frac{4a+a'}{R^2} & \boldsymbol{y} \in A, \\ k\lambda & \boldsymbol{y} \in B. \end{cases}$$

Note, that under the assumptions discussed above, the Lagrange multiplier λ has a direct physical interpretation of the tension in the suspended region τ_B normalized by the membrane stiffness k.

Tension dependency on the angle The free energy (7) due to the constraint (9) has only one free parameter, the wetting angle θ. Consequently, the tension in the envelope in the equilibrium also depends solely on the wetting angle. Moreover, the tension depends on the wetting angle only via the Lagrange multiplier λ, which itself is given by constraint (9). Thus we can apply the rule for the derivative of the implicit function,

$$\partial_\theta \tau(\boldsymbol{y}) = k\partial_\theta \lambda = -k\frac{\partial_\theta g}{\partial_\lambda g} = \frac{\pi k R^2(\lambda+1)\sin\theta \left[\frac{k^2(\lambda+1)^2}{[k(\lambda+1)-\varepsilon'(R)]^2} - \cos\theta\right]}{d^2 - \pi R^2 \sin^2\theta + 2\pi R^2 (1-\cos\theta)\frac{k^3(\lambda+1)^3}{[k(\lambda+1)-\varepsilon'(R)]^3}}.$$

Note, that $k(\lambda+1) > \varepsilon'(R) \geq 0$, cf. (5), thus the sign of the derivative is solely determined by the sign of the wetting angle θ. We can conclude that the tension and bactericidal activity always increases with the magnitude of the wetting angle $|\theta|$, see figure 4. Because the free energy and the constraint is even with respect to the wetting angle, $\mathcal{F}(-\theta) = \mathcal{F}(\theta)$ and $g(\theta) = g(-\theta)$, we can further restrict our analysis only to the non-negative wetting angle, $\theta \geq 0$. This result can further imply that the tension in the envelope is maximal for maximally covered pillars, i.e. $\theta \equiv \theta_{\max} = \arcsin \rho/R$.

3.1 Large adhesion regime

In previous section we observed that for sufficiently large adhesion energy density the free energy is minimized for the envelope completely covering the pillar caps, $\theta \equiv \theta_{\max}$. We will further investigate this particular regime here and see which topography maximizes the tension of the cell's envelope, that can lead to the rupture of the envelope and consequently to the lysis. In other words an optimal bactericidal surface would be a surface maximizing the tension of the cell's envelope.

We assume that the wetting angle is maximal, $\theta \equiv \theta_{\max}$. This simplifies the expression for the free energy (7) and the constraint (9) even further to

$$\begin{aligned} \mathcal{F} &= k\frac{\left[k^2\lambda^2 - (2\lambda+1)k\varepsilon'(R) + \varepsilon'(R)^2\right]\left[2\pi R^2\left(1-\sqrt{1-\frac{\rho^2}{R^2}}\right)\right]}{\left[k(\lambda+1)-\varepsilon'(R)\right]^2} + \frac{k\lambda^2\left[d^2-\pi\rho^2\right]}{(\lambda+1)^2}, \\ d^2 &= \frac{k^2\left[2\pi R^2\left(1-\sqrt{1-\frac{\rho^2}{R^2}}\right)\right]}{\left[k(\lambda+1)-\varepsilon'(R)\right]^2} + \frac{\left[d^2-\pi\rho^2\right]}{(\lambda+1)^2}, \end{aligned} \tag{11}$$

where the second equation now fully determines the Lagrange multiplier λ and consequently the free energy $\mathcal{F}$.

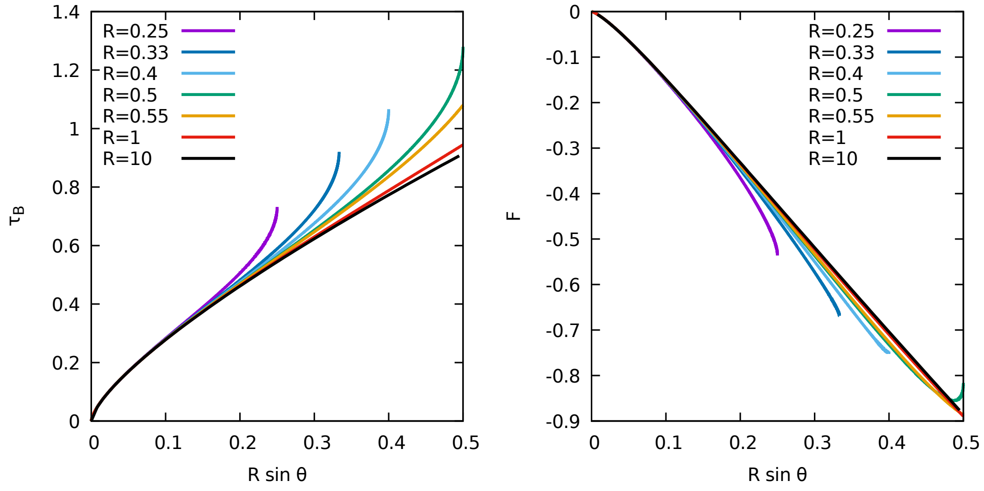

Figure 4: Dependency of the tension in the envelope in the suspended region τ_B and corresponding free energy on the wetting angle θ for various radii of curvature R for the envelope without any bending rigidity $a = a' = 0$. Other parameters are assumed to be $k = 1$, $d = 1$ and $\varepsilon = 1$. We observe that for arbitrary curvature the tension always increases with the wetting angle. Also note that increase of the tension induced by the adhesion indeed minimizes the free energy.

Adhesion energy influence We first evaluate the influence of the adhesion energy density on the tension. In particular, we need to compute the derivative

$$\partial_\varepsilon \tau(\boldsymbol{y}) = k\partial_\varepsilon \lambda - \chi_A(\boldsymbol{y}) = -k\frac{\partial_\varepsilon g(\lambda, \theta_{\max})}{\partial_\lambda g(\lambda, \theta_{\max})} - \chi_A(\boldsymbol{y}) = \frac{1}{1 + \frac{S_B}{S_A}\left[1 - \frac{\varepsilon'(R)}{k(1+\lambda)}\right]^3} - \chi_A(\boldsymbol{y}),$$

where $\chi_A(\boldsymbol{y})$ is a characteristic function of the adhered region A, $\chi_A(\boldsymbol{y}) = 1$ for $\boldsymbol{y} \in A$ and $\chi_A(\boldsymbol{y}) = 0$ elsewhere, S_A is the area of the envelope in the adhered region and S_B is the area of the envelope in the suspended region,

$$S_A = 2\pi R^2 \left(1 - \sqrt{1 - \frac{\rho^2}{R^2}}\right), \qquad S_B = d^2 - \pi\rho^2.$$

As $k(\lambda + 1) > \varepsilon'(R)$, cf. (5), the tension in the envelope decreases with increased adhesion strength in case the envelope is in contact with the surface and increases if it is not,

$$\begin{aligned} \partial_\varepsilon \tau(\boldsymbol{y}) &\leq 0 \quad \text{for} \quad \boldsymbol{y} \in A, \\ \partial_\varepsilon \tau(\boldsymbol{y}) &\geq 0 \quad \text{for} \quad \boldsymbol{y} \in B, \end{aligned} \tag{12}$$

see figure 5. This means that increasing the adhesive properties of the surface shall lead to higher bactericidal activity.

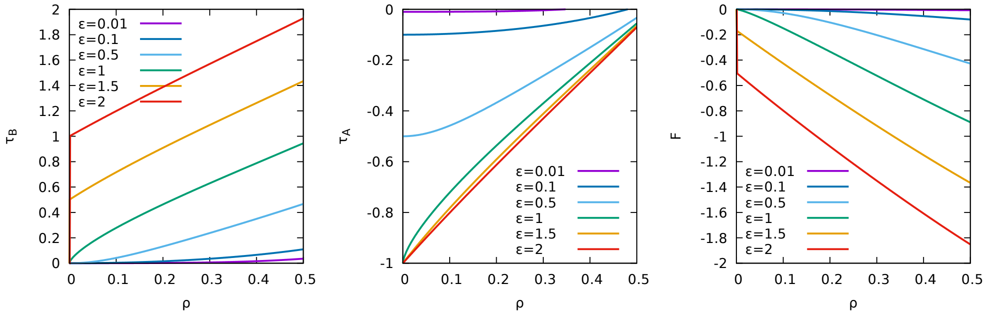

Figure 5: Dependency of the tension in the envelope in the suspended region τ_B, the adhered region τ_A and corresponding free energy on the pillar radii ρ for various adhesion energies ε. Here we consider an envelope without any bending rigidity $a = a' = 0$ only, with other parameters given by $k = 1$, $d = 1$ and $R = 1$. We further observe that the tension in the adhesive and suspended region has universally opposite tendencies as a function of the adhesion energy.

Pillar's cap radius Analogous to the previous case we derive that the tension increases with the pillar's cap radius ρ

$$\partial_\rho \tau(\boldsymbol{y}) = k\partial_\rho \lambda = -k\frac{\partial_\rho g(\lambda, \theta_{\max})}{\partial_\lambda g(\lambda, \theta_{\max})} = \pi\rho\left[k(\lambda+1) - \varepsilon'(R)\right] \frac{\frac{1}{\sqrt{1-\frac{\rho^2}{R^2}}} - \left[1 - \frac{\varepsilon'(R)}{k(\lambda+1)}\right]^2}{S_A + S_B\left[1 - \frac{\varepsilon'(R)}{k(\lambda+1)}\right]^3} > 0.$$

Note that the derivative diverges for $\rho \to R$, which can be seen in figure 4.

The dependency of the tension on the pillar's cap radius for the flexible envelope $a \approx a' \approx 0$ is the weakest for flat cylinders $R \to \infty$,

$$\partial_\rho \tau(\boldsymbol{y})|_{R\to\infty} = \frac{1}{\rho}\left[k(\lambda+1) - \varepsilon\right] \frac{1 - \left(1 - \frac{\varepsilon}{k(\lambda+1)}\right)^2}{1 + \frac{S_B}{S_A}\left[1 - \frac{\varepsilon}{k(\lambda+1)}\right]^3} < \partial_\rho \tau(\boldsymbol{y})|_{R=\text{finite}},$$

see figure 6. We further observe that for moderate bending rigidity a sufficiently large pillar's cap radius is necessary for the curvature to induce higher tension, and thus improve bactericidal properties of the surface, while in the case of the large bending rigidity and sufficiently larger curvature the surface became biofouling regardless of the pillar's cap radius.

Cap's curvature Using the same methodology as before, we investigate the influence of the radius of curvature on the tension,

$$\begin{aligned}\frac{\mathrm{d}\tau(\boldsymbol{y})}{\mathrm{d}R} &= \partial_\varepsilon \tau(\boldsymbol{y})\,\partial_R \varepsilon'(R) - k\frac{\partial_R g(\lambda, \theta_{\max})}{\partial_\lambda g(\lambda, \theta_{\max})} \\ &= 2\frac{4a + a'}{R^3}\partial_\varepsilon \tau(\boldsymbol{y}) + \frac{\pi R}{\sqrt{1 - \frac{\rho^2}{R^2}}} \frac{2\sqrt{1 - \frac{\rho^2}{R^2}} - 2 + \frac{\rho^2}{R^2}}{S_A + S_B\left(1 - \frac{\varepsilon'(R)}{k(\lambda+1)}\right)^3}\left[k(\lambda+1) - \varepsilon'(R)\right]. \qquad (13)\end{aligned}$$

From (12) it immediately follows that the first term is negative for the envelope adhering to the surface, $\boldsymbol{y} \in A$, and positive otherwise. Regarding the second term, its second fraction's

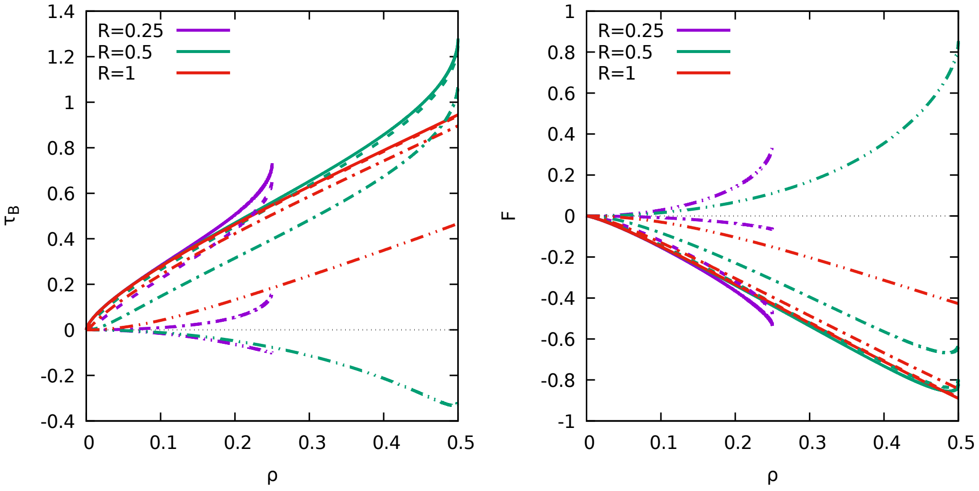

Figure 6: Dependency of the tension in the envelope in the suspended region τ_B and corresponding free energy on the pillar's cap radius ρ for various radii of curvature R and bending rigidities a. The solid line denotes $a = 0$, dashed line $a = 2 \times 10^{-4}$, dot-dashed line $a = 2 \times 10^{-3}$ and double-dot-dashed line $a = 2 \times 10^{-2}$. Other parameters are assumed to be $a' = a$, $k = 1$, $d = 1$. We observe that for moderate bending rigidity larger curvature induces higher tension only for sufficiently large pillar's cap radius, while in the case of the large bending rigidity the surface with larger curvature became non-adhesive, i.e. biofouling.

numerator determines the overall sign of the second term as all the other expressions are positive. From the relation between the arithmetic and the geometric mean, $\sqrt{1 \cdot (1+x)} \leq (1 + (1 + x))/2 = 1 + x/2$, it directly follows that the given numerator and thus the second term is always non-positive. This means that the tension increases with curvature (decreases with radius of curvature) uniformly in the adhered region of the envelope. However, the dependency of the tension in the envelope suspended between caps on the curvature is non-trivial and is determined by the competition between the first term representing the bending rigidity and the second term representing the increase of adhesive area. This competitive effect is illustrated in figure 6, where for the envelope with the zero bending rigidity the tension uniformly increases with the curvature, while for the envelope with intermediate bending rigidity the trend is no longer monotonous and depends on the radius of the pillars' caps. This is further demonstrated in figure 7.

Spacing At last, we investigate the influence of the spacing d on the tension. By using the (5) and by assuming $\lambda > 0$ we obtain

$$\partial_d \tau(\boldsymbol{y}) = k\partial_d\lambda = -k\frac{\partial_d g(\lambda,\theta_{\max})}{\partial_\lambda g(\lambda,\theta_{\max})} = -\frac{kd\lambda\,(\lambda+1)\,(\lambda+2)}{S_A}\frac{\left[1-\frac{\varepsilon'(R)}{k(1+\lambda)}\right]^3}{1+\frac{S_B}{S_A}\left[1-\frac{\varepsilon'(R)}{k(1+\lambda)}\right]^3} \leq 0.$$

Thus tighter packing increases globally the tension in the envelope and consequently the bactericidal activity. See figure 8 for illustration.

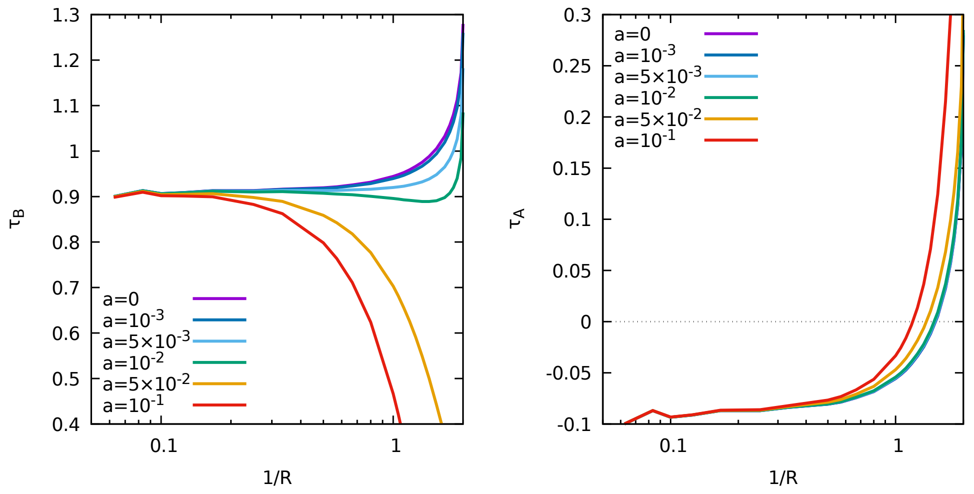

Figure 7: Dependency of the tension in the envelope in the adhered region τ_A and suspended region τ_B on the curvature $1/R$ for various bending rigidities. Other parameters are assumed to be $a' = a$, $k = 1$, $d = 1$, $\varepsilon = 1$ and $\rho = 0.5$. We observe that for moderate bending rigidity, a larger curvature induces higher tension, while in the case of a large bending rigidity a surface with large curvature became biofouling.

Deposition	0 nm	85 nm	100 nm	130 nm
ρ_{Otsu}	17.5 nm	40.1 nm	42.3 nm	45.7 nm
ρ_{fit}	17.5 nm	41.0±1.7 nm	46.2±2.9 nm	*44.6±8.1 nm*
R	∞	41.5±1.5 nm	47.0±3.2 nm	*49.0±11.7 nm*
$s_A^{\max}$	12 %	112.9 %	139.9 %	*109.1 %*
$s_B^{\min}$	88 %	37.7 %	30.5 %	19.0 %

Table 1: Estimated pillar cap radius ρ and curvature radius R for various gold deposition thicknesses.Corresponding estimates of a maximal and minimal relative areas s_A, s_B. By italic font we denote those values where the fitting procedure failed to converge.

4 Model validation

To validate the proposed model we want to estimate the tension, we will try to estimate adhesion induced tension in a bacterial envelope on the manufactured surfaces. In order to achieve this goal we will first estimate the geometry of the manufactured gold sputtered surface. Second, we discuss typical mechanical parameters of a bacterial envelope. Finally, we estimate the tension induced by these surfaces on a typical bacterial envelope for various degree of adhesion.

4.1 Geometrical parameters

All surfaces were prepared with the pitch of $d = 90\,\text{nm}$. By using the top view image of each of the surfaces obtained by scanning electron microscope (SEM), we can determine the relative area the pillars occupy a_A with the Otsu's method [20], see figure 9. From the relative area a_A we can also estimate the radius of the spherical cap as $\rho_{\text{Otsu}} = d\,(a_A/\pi)^{1/2}$ and the minimal relative area of the suspended membrane $s_B^{\min} = 1 - a_A$. The values are summarized in table 1.

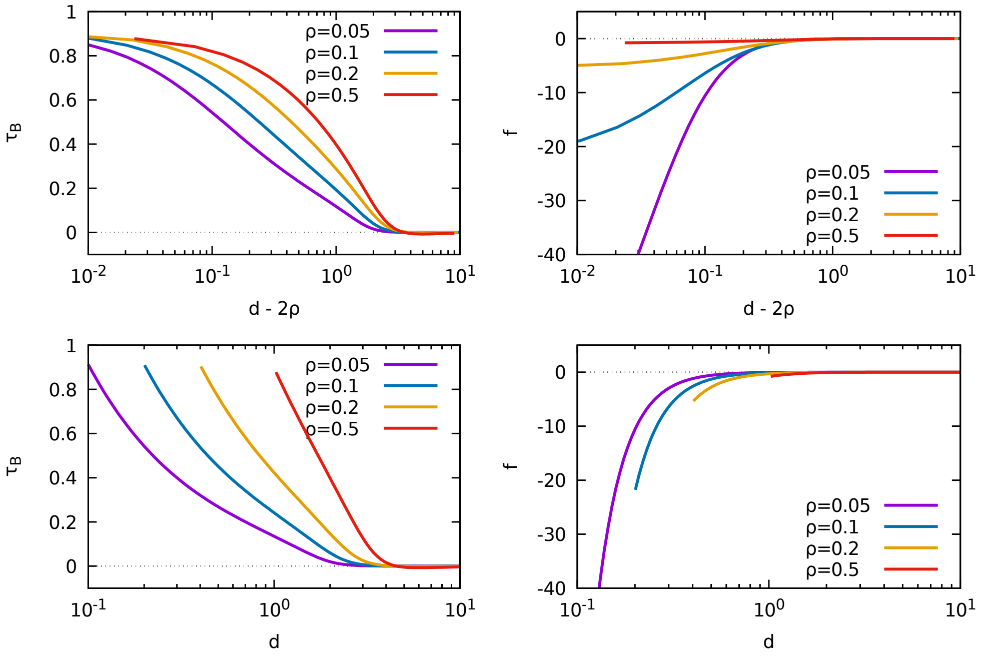

Figure 8: Dependency of the tension in the envelope in the suspended region τ_B and free energy density f on the gap size $d - 2\rho$ and center-to-center distance d for various pillar radii. Other parameters are assumed to be $a' = a = 10^{-2}$, $k = 1$, $R = 1$ and $\varepsilon = 1$. We observe that a larger radius of pillar's caps induce higher tension for both equal spacing and equal gap. We also observe that in case of equally spaced pillars, the envelope preferentially adheres to those the with larger caps. On the other hand, for pillars separated by equal gap, the envelope adheres better to smaller pillar caps and thus reduces both tension and bactericidal activity.

We further fitted spherical caps using least square method, where the pixel's value was interpreted as an intensity of the beam reflected from the spherical cap according to Lambert's cosine law. Namely,

$$\min_{\{\boldsymbol{x}_i, R_i, \rho_i, I_i\}} \sum_p \left| I_p - \sum_{i : \|\boldsymbol{x}_p - \boldsymbol{x}_i\| \le \rho_i} f_i(\boldsymbol{x}_p) \right|^2,$$

where

$$f_i(\boldsymbol{x}) = \begin{cases} I_i \sqrt{1 - \frac{\|\boldsymbol{x}-\boldsymbol{x}_i\|^2}{R_i^2}} & \|\boldsymbol{x} - \boldsymbol{x}_i\| \le R_i, \\ 0 & \text{otherwise} \end{cases}$$

and where the minimization is over all possible positions $\boldsymbol{x}_i$ of all spherical caps i, caps radii ρ_i, caps curvature radii R_i and reflected beam intensity I_i. The index p denote individual pixels with positions $\boldsymbol{x}_p$ and intensities I_b. In order to improve the stability of the fitting procedure, we removed the noise from the image in the suspended region by using the image processed with the Otsu's method as a mask on the original gray-scale image (see figure 10 left). Moreover, we

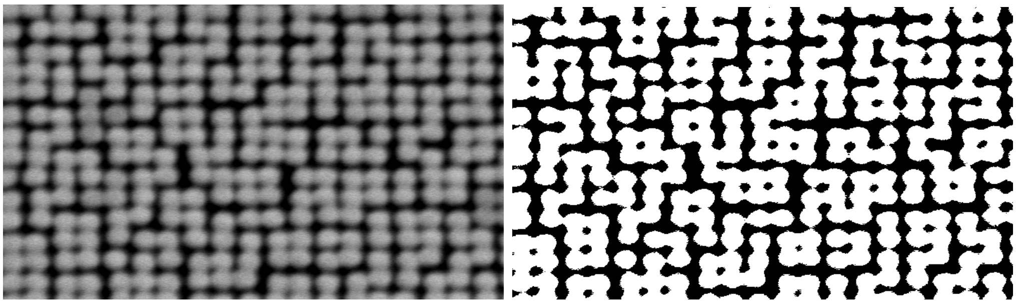

Figure 9: Original gray scale image with top view of the surface for 85 nm thick deposition (left) and a processed image with Otsu's method (right). The relative area a_A is then determined as a number of white pixels in the processed image with respect to the total number of all pixels.

further extended the fitted function f_i to

$$\tilde{f}_i(\boldsymbol{x}) = \begin{cases} I_i\sqrt{1-\frac{\|\boldsymbol{x}-\boldsymbol{x}_i\|^2}{R_i^2}} & \|\boldsymbol{x}-\boldsymbol{x}_i\| \le R_i, \\ -I_i\sqrt{1-\left(2-\frac{\|\boldsymbol{x}-\boldsymbol{x}_i\|}{R_i}\right)^2} & R_i < \|\boldsymbol{x}-\boldsymbol{x}_i\| \le 2R_i, \\ -I_i & \text{otherwise.} \end{cases}$$

This allow us to gradually increase a penalisation with increasing cap radius ρ in case the perceived pillar radius is larger than radius of curvature, $\rho > R$, and thus improve the stability of the least square method. See figure 10 for the example of the initial image, final image and the absolute error of the fitting method. The values of spherical cap radius ρ_{fit} and radius of curvature R found by the fitting procedure are also summarized in table 1. Note that, the fitting procedure failed to converge for 130 nm thick deposition, as the spherical caps no longer reliably describe the surface geometry. For this reason, we omit the values of 130 nm from further analysis. Knowing these values, we can further estimate the maximal relative area $s_A^{\max}$ as

$$s_A^{\max} = 2\pi\left(\frac{R}{d}\right)^2\left(1-\sqrt{1-\frac{\rho_{\text{fit}}^2}{R^2}}\right).$$

4.2 Bacterial envelope physical parameters

Before we can evaluate the induced tension, we need to determine the relevant physical parameters of the cell envelope. Namely, we need to determine cell envelope stiffness k, adhesion density ε and bending energy density a. As the cell envelope is a heterogeneous, anisotropic, visco-elastic system consisting of multiple layers, which chemical and physical characteristics greatly vary from species to species, it is impossible to assign a individual numbers to these parameters. Nonetheless, as we are interested only in qualitative agreement between our model and experiments, the order of magnitude along with the interval estimates shall suffice.

Adhesion Katsikogianni *et al.* determined the Gibbs free energy density of the *Staphylococcus epidermis* adhering to alkyl silan layer deposited on the glass from the LW-AB theory in the range of $\Delta g_{\text{adh}} = -10\,\text{pN}\,\text{nm}^{-1}$ to $-80\,\text{pN}\,\text{nm}^{-1}$, [16, 17]. Similarly, Busalmen and Sánchez [5] used DLVO theory to estimate adhesion of *Pseudomonas fluorensis* on gold and found primary minimum in the DLVO potential exceeding $1800\,k_BT$, which is comparable with minimums around

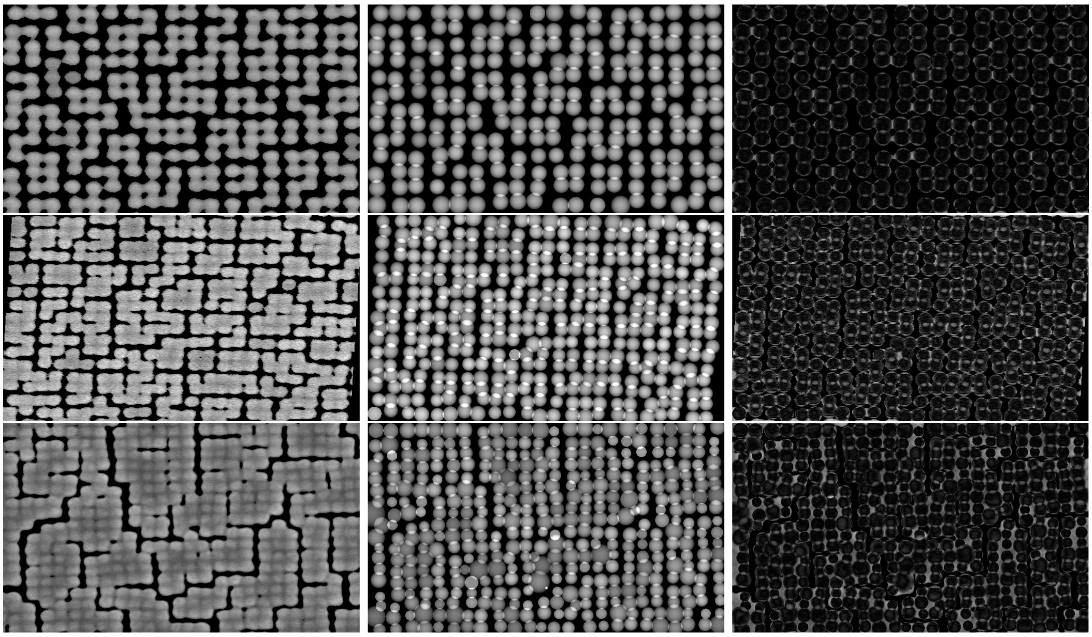

Figure 10: The masked gray scale image (left), fitted spherical caps (middle) and the absolute difference between the masked image and fitted spherical caps (right) using the same gray scale for the surface with deposition thicknesses of 85 nm, 100 nm and 130 nm (from top).

1600 $k_B T$ reported in [17]. An alternative and a more direct approach is able to link the reported maximal pull-of forces in the range of -1.5 nN to -3 nN (values obtained for *E. coli* adhering to geothite [6, 12]) to the adhesion energy density using the Brochard-Wyart and de Gennes theory [24]. This leads to the adhesion energy density in the range $\varepsilon = 1\,\mathrm{nN\,\mu m^{-1}}$ to $2\,\mathrm{nN\,\mu m^{-1}}$ with the characteristic radius estimated to be $\hat{R} = 0.5\,\mathrm{\mu m}$. Another approach is surface-enhanced-fluorescence to estimate deformations during the adhesion. Li *et al.* [18] found deformations of the *Staphylococcus aureus* cells in the range from 125 nm to 175 nm and deformations of cell walls alone around 20 nm after 3 h exposition to a gold surface. By using these values, the contact area and from the standard Hertz theory also the energy of the deformation induced by adhesion can be estimated. This lead to adhesion energy density around $60\,\mathrm{pN\,nm^{-1}}$. Using all the estimates to adhesion energy together gives us a range of the adhesion energy density $\varepsilon \approx 1\,\mathrm{pN\,nm^{-1}}$ to $100\,\mathrm{pN\,nm^{-1}}$, whereas values for matured adhesions for both *Pseudomonas aeruginosa* and *Staphylococcus aureus* are expect to be close to the upper bound.

Stiffness The cell envelope is composite material consisting of one or more cell membranes and a single cell wall. Hwang *et al.* [13] estimated the stiffness of inner and outer membrane of *E. coli* with the means of a molecular dynamics simulation, they found the values of $k_I \approx 200\,\mathrm{mN\,m^{-1}}$ in the linear regime for inner membrane and $k_O \approx 530\,\mathrm{mN\,m^{-1}}$ for outer membrane. Moreover, they estimated the critical tension leading to a rupture $\tau_{\mathrm{crit}} \approx 75\,\mathrm{mN\,m^{-1}}$ for an inner membrane, which is in good agreement with previously experimentally estimated values between $30\,\mathrm{mN\,m^{-1}}$ and $60\,\mathrm{mN\,m^{-1}}$ [10]. They also experimentally estimated the stiffness of the cell wall $k_W \approx 15\,\mathrm{mN\,m^{-1}}$ to $1370\,\mathrm{mN\,m^{-1}}$, depending on the turgor pressure. And consequently, they determined that the inner and outer membrane bear anywhere from approximately 33 % down to 10 % of the load each, while the cell wall bears 35 % up to 78 % of the load under the

Parameter		Symbol	Value/Range
Adhesion energy density	- low	ε_L	$1\,\mathrm{pN\,nm^{-1}}$
	- high	ε_H	$100\,\mathrm{pN\,nm^{-1}}$
Cell envelope stiffness	- low	k_L	$500\,\mathrm{mN\,m^{-1}}$
	- high	k_H	$1690\,\mathrm{mN\,m^{-1}}$
Load distribution	- low		$35\,\%$
	- high		$11\,\%$
Critical tension		τ_{crit}	$30\,\mathrm{mN\,m^{-1}}$ to $75\,\mathrm{mN\,m^{-1}}$
Bending rigidity	- low	a_L	$40\,k_{\mathrm{B}}T$
Normalized adhesion energy density	- low + high	$\hat{\varepsilon}_{LH}$	0.6×10^{-3}
	- low + low	$\hat{\varepsilon}_{LL}$	2×10^{-3}
	- high + high	$\hat{\varepsilon}_{HH}$	59×10^{-3}
	- high + low	$\hat{\varepsilon}_{HL}$	200×10^{-3}
Normalized critical tension	- low	$\hat{\tau}_L$	0.17 to 0.43
	- high	$\hat{\tau}_H$	0.16 to 0.40
Normalized bending rigidity	- low + high	$\hat{a}_{LH}$	$0.1\,\mathrm{nm}^2$
	- low + low	$\hat{a}_{LL}$	$0.3\,\mathrm{nm}^2$
	- high	$\hat{a}_H$	$64\,\mathrm{nm}^2$

Table 2: Summary of typical values and ranges of various bacteria envelope parameters and their combinations to normalized parameters, $\hat{A}_{XY} = A_X/k_Y$.

same turgor pressure [13]. The overall stiffness of the cell envelope was found to be in the range of$k \approx 500\,\mathrm{mN\,m^{-1}}$ to $1690\,\mathrm{mN\,m^{-1}}$.

Bending rigidity Although, it is well know that the bending rigidity of the lipid bi-layer is $a_{\text{bi-layer}} \approx 20k_BT$ [1, 2, 21], the bending rigidity of the cell envelope is much less understood. As gram-negative bacteria has two lipid bi-layers, if we consider them independent we will obtain the lower bound on the bending rigidity $a \approx 40k_BT$. Nonetheless, the real bending rigidity of the composite material can be much larger then this estimate due to bonding between individual layers. A rough estimate of the upper bound we can obtain if we apply the theory of bending rigidity of thin homogeneous sheets [8], which assumes a dense homogeneous sheet, or in another word in our setup assumes perfect coupling between individual layers. Within this theory the bending rigidity of the sheet is linked to its stiffness by

$$a_H = \frac{kh^2}{12\left(1-\nu^2\right)},$$

where $h = 24\,\mathrm{nm}$ is the envelope thickness [13], $\nu = \frac{1}{2}$ is its Poisson ratio [13]. Note, that the normalized bending rigidity does not depend on the stiffness

$$\hat{a}_H = \frac{a_H}{k} = \frac{h^2}{12\left(1-\nu^2\right)} = 64\,\mathrm{nm}^2.$$

Moreover, for the lack of other data we will further assume $a = a'$.

All the parameters discussed above are summarized in table 2.

4.3 Induced tension

By using the iterative scheme described in appendix A we can evaluate the induced tension in the situation when bacteria adhere to a surface. As most of the parameters have range

ε	k	a	$\hat{\varepsilon}' \times 10^3$ —	85 nm	100 nm	$\hat{\tau}_{\max} \equiv \lambda_{\max}$ 0 nm	85 nm	100 nm
L	H	H	0.6	−142	−110	$7.2{\times}10^{-5}$	—	—
L	H	L	0.6	0.31	0.37	$7.2{\times}10^{-5}$	0.23	**0.31**
L	L	H	2	−140	−109	$2.4{\times}10^{-4}$	—	—
L	L	L	2	1.13	1.32	$2.4{\times}10^{-4}$	0.23	**0.31**
H	H	H	59	−83	−52	$7.6{\times}10^{-3}$	—	—
H	H	L	59	58.71	58.77	$7.6{\times}10^{-3}$	0.27	**0.35**
H	L	H	200	57.74	89.09	$3.2{\times}10^{-2}$	0.27	**0.38**
H	L	L	200	199.13	199.32	$3.2{\times}10^{-2}$	**0.38**	**0.47**

Table 3: Reduced effective adhesion energy and induced membrane tension at maximal coverage. Dash marks configuration of parameters where the cell does not adhere to the surface, while bold font marks highly bactericidal configurations with induced tension values in the upper half of the critical tension range, $\hat{\tau} > 0.3$. Note, that the resulting normalized tensions correspond to an actual induced tension $\tau \approx 115\,\mathrm{nN\,\mu m^{-1}}$ to $590\,\mathrm{nN\,\mu m^{-1}}$ for surfaces with deposition while only to $\tau \approx 0.12\,\mathrm{nN\,\mu m^{-1}}$ to $16\,\mathrm{nN\,\mu m^{-1}}$ for the surface without deposition.

spanning several order of magnitudes, we opted to evaluate the induced tension for all possible combinations of low and high bounds of each interval. These values are listed in the table 3 along with the normalized effective adhesion for all possible combinations of lower (L) and upper (H) bounds of adhesion, membrane stiffness and bending rigidity. All values of these parameters are listed in table 2 along with their normalized variants, $\hat{A}_{XY} = A_X/k_Y$.

These results suggest that the manufactured surface without any deposition shall not be bactericidal at all as all the possible induced tension is at least one order of magnitude smaller then the minimal threshold value $\hat{\tau}_{\mathrm{crit}}^{\min} = 0.16$. This result is consistent with our observation where the untreated manufactured surface didn't exhibit any bactericidal activity. For the surface with 85 nm thick deposition the model suggests two possible scenarios. First, for large bending rigidity of the bacteria and low to moderate adhesion the surface become biofouling, see also figure 2. While in all other cases the induced tension shall be large enough for the surface to be bactericidal. A similar result is obtained for the surface with 100 nm thick deposition, although the values of the induced tension are much closer or are even exceeding to the upper bound of the critical tension $\hat{\tau}_{\mathrm{crit}}^{\min} = 0.41$. This suggest that the surface with 100 nm thick deposition layer shall be more bactericidal then the surface with 85 nm thick deposition. Indeed, in the figure 8 of the main text shows that the surface with 100 nm thick deposition layer has almost an order of magnitude higher bactericidal activity then the surface with only 85 nm thick deposition.

Note, that the values described in the table are provided under the assumption that the membrane in the spanning region is flat, which would lead to unrealistic sharp angles formed at the membrane. However, contribution from this additional curvature only increases the induced tension and is limited to a small region close to the edge of the pillar.

4.4 Wetting angle

Up to this point we always discussed the situation where the bacteria envelope adheres the all available surface of the nanopillar cap. In order to find out a realistic wetting angle we numerically evaluated the free energy function (11) and normalized tension $\hat{\tau}$ as a function of the cap's radius of curvature relative to the pitch distance $\frac{R}{d}$ and wetting angle θ, represented as pillar's cap radius $\rho = R\sin\theta$, for various level of normalized effective adhesion $\hat{\varepsilon}'$, see figure 11. Note, that in reality the curvature influences also the normalized effective adhesion $\hat{\varepsilon}'$ via the

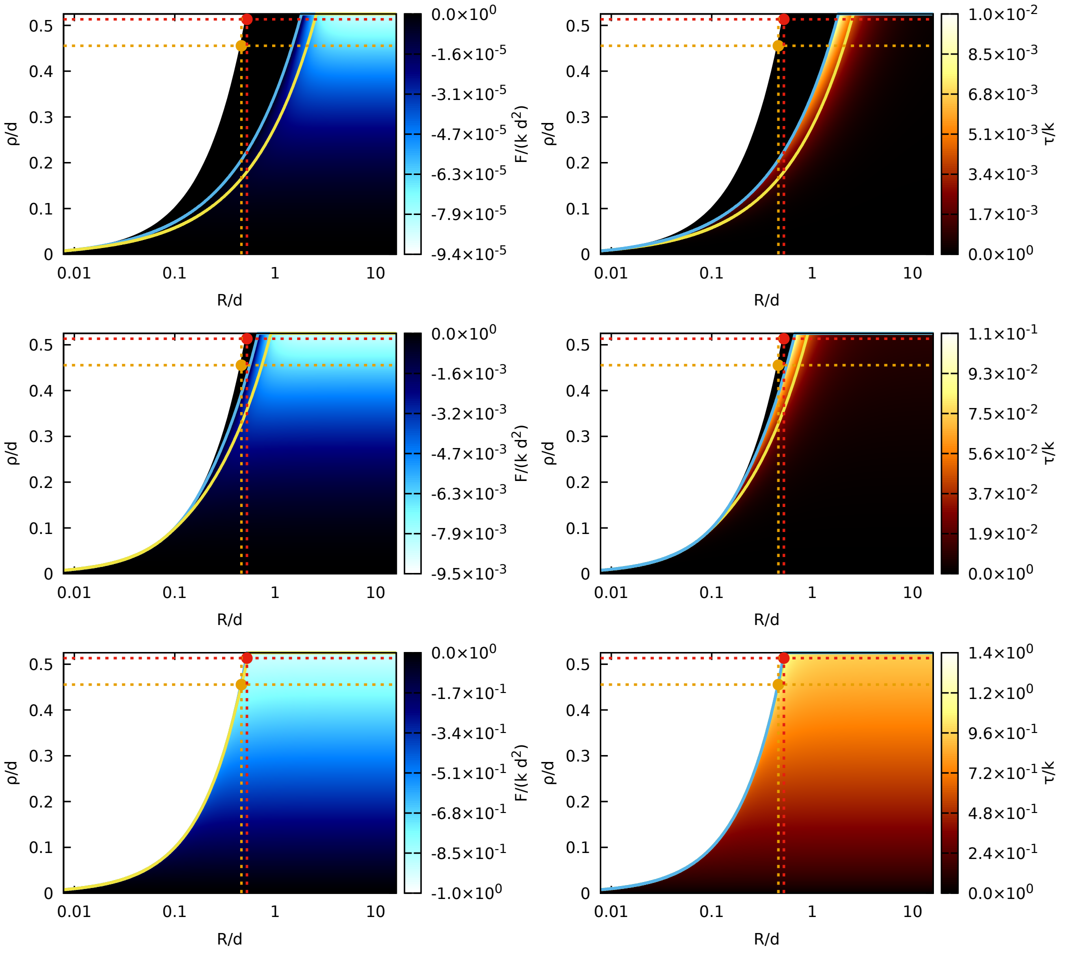

Figure 11: The free energy landscape (left column) and normalized induced tension (right column) for various levels of normalized effective adhesion ($\hat{\varepsilon}' \in \{10^{-4}, 10^{-2}, 1\}$ from the top) as a function of the relative curvature $\frac{R}{d}$ and wetting angle θ represented as a relative pillar's cap radius $\frac{\rho}{d} = \frac{R}{d}\sin\theta$. Yellow line determines the minimum of the free energy for the given curvature, while blue line determines the maximal induced tension. Orange (resp. red) dotted lines then correspond to the observed curvature radius R and pillar's cap radius ρ for 85 nm (resp. 100 nm) thick deposition of gold. Note, that the maximal tension corresponds to the situation, where the bacteria loose the ability to further adhere to the surface, $\mathcal{F} = 0$.

bending rigidity. However, this would expand significantly a set of possible parameters, so we simplified the analysis by assuming $a = a' = 0$.

We observe that free energy $\frac{\mathcal{F}}{kd^2}$ in general decreases with the increasing wetting angle, while the normalized tension $\hat{\tau}$ increases. Although, there exists a range of relative radii of curvature $\frac{R}{d}$ for low normalized effective adhesion $\hat{\varepsilon}' \in \{10^{-4}, 10^{-2}\}$, where the behaviour is non-monotonous. This confirm a general trend that for a given cap's radius of curvature the surface with larger pillars is more bactericidal.

In particular, for pillars with large curvatures (small $\frac{R}{d}$) the cell envelope tend to cover the

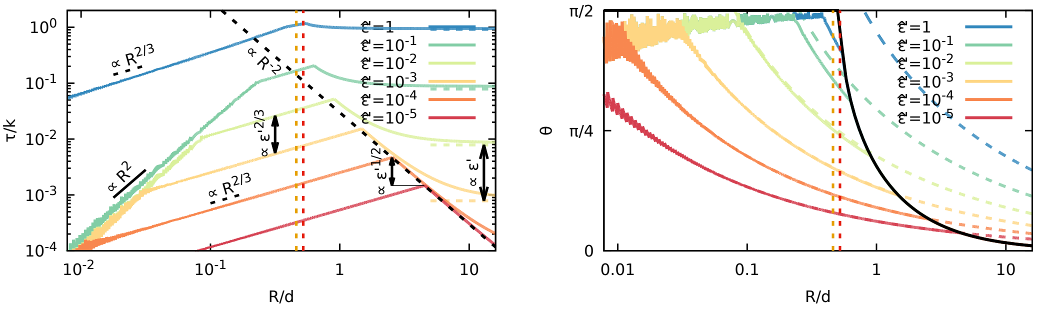

Figure 12: The normalized tension $\frac{\tau}{k}$ and wetting angle θ at minimum of the free energy as a function of a relative curvature $\frac{R}{d}$ for various levels of normalized effective adhesion $\hat{\varepsilon}'$. We observe three distinct regimes in tension dependency on the curvature. In the first regime, which corresponds to $\hat{\tau} \ll \hat{\varepsilon}'$, the tensions increases as $\hat{\tau} \propto R^2$ as the minimum of the free energy follows $\rho = R$. In the second regime we depart from the maximal possible cover, $\rho < R$, and the tension scales as $\hat{\tau} \propto R^{\frac{2}{3}}$. The last regime correspond to situation when geometry of the surface limit the maximal size of the pillars, $\frac{\rho}{d} \leq \frac{1}{2}$. Consequently, the induced tension relaxes with increasing radius of curvature toward the induced tension of the flat pillar of equivalent size. The local maxima of induced tension represent the optimal bactericidal configuration for the given level of the effective adhesion. We summarized these optimal configurations into the table 1 of the main manuscript. Note, that the $\hat{\varepsilon}' = 1$ has an anomalous trend of $\hat{\tau} \propto R^{\frac{2}{3}}$ even in the first regime, see appendix A.1 for further details.

pillars cap completely as the minimum of the free energy (yellow line in figure 11 is located as $\theta = \frac{\pi}{2}$, see also figure 12. Moreover, the minimum of the free energy for a given relative radius of curvature $\frac{R}{d}$ coincide with the maximum of induced tension (blue curve in figure 11).

For the intermediate curvature range, the minimum of the free energy departs from the maximal wetting conditions as well as from the maximum of the induced tension. This also mean that increasing the pillar's cap radius beyond the one given by the minimum of the free energy $\rho > \rho(\mathcal{F}_{\min})$ won't improve the bactericidal activity anymore. By investigating the normalized induced tension as function of relative radius of curvature $\frac{R}{d}$, we have found out that transition between this regime and high curvature regime occurs when the normalized induced tension is equal to the normalized effective adhesion, $\hat{\tau} \approx \hat{\varepsilon}'$, see figure 12. We have found out that while in the first regime the tension at the minimum of the free energy scales with the radius of curvature as $\hat{\tau} \propto R^2$ independently of the value of the normalized effective adhesion, see appendix A.1, in the second regime the normalised tension for a given normalized effective adhesion energy scales as $\hat{\tau} \propto R^{\frac{2}{3}}$. We have shown that in this regime the normalized tension also depends on the normalized effective adhesion as $\hat{\tau} \propto \hat{\varepsilon}'^{\frac{2}{3}}$ and the optimal pillar's cap radius depend on the radius of curvature as $\rho \propto R^{\frac{1}{3}}$, see appendix A.2 and figure 12 for further details.

As the optimal pillar's cap radius ρ increases with increasing radius of curvature R we get to a situation with tightly packed pillars, $\rho = \frac{d}{2}$. Since the pillar's cap radius is capped by this value, as we cannot physically place larger pillars on the grid, further increase in the radius of curvature R lead to suboptimal wetting angle, $\theta \leq \theta(\mathcal{F}_{\min})$. Consequently, in this regime, the tension decreases towards flat pillar asymptotic, see colored dashed lines figure 12 and appendix A.3.

Note, that the transition point between second and third regime corresponds to the maximum of the normalized tension, and thus represent the configuration with the maximal bactericidal activity. Moreover, for small normalized effective adhesions $\hat{\varepsilon}' \ll 1$ we were able to determine

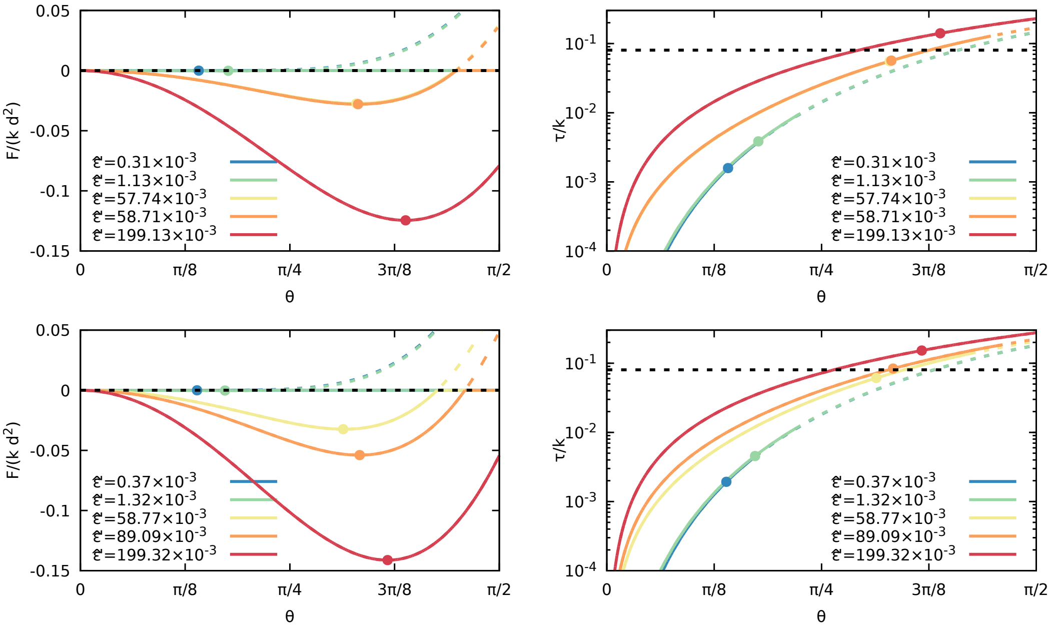

Figure 13: Normalized free energy $\frac{\mathcal{F}}{kd^2}$ (left) and normalized tension $\hat{\tau}$ as a function of wetting angle for pillars with deposition thickness 85 nm (top) and 100 nm (bottom) for various level or normalized effective adhesion $\hat{\varepsilon}'$. Colored dashed lines are continuations beyond the point where adhesion is favorable $\mathcal{F} > 0$. Black dashed line represent $\mathcal{F} = 0$ and $\hat{\tau} = \hat{\tau}_{\mathrm{crit}}$ respectively.

the maximal normalized induced tension as

$$\hat{\tau}_{\max} \approx \frac{1}{4}\sqrt{\pi\hat{\varepsilon}'}$$

for pillars with radius of the cap $\rho = \frac{d}{2}$ and radius of curvature

$$R_{\max} \approx \sqrt[4]{\frac{\pi}{2^{10}\hat{\varepsilon}'}}\, d,$$

see appendix A.2 for detailed derivation. In practice, the last relation is non-trivial as normalized effective adhesion depends on the radius curvature due to the bending rigidity of the bacterial envelope.

These results suggest that for the manufactured geometry of the pillars the wetting angle might play an important role in the induced tension, and consequently in bactericidal activity. In order to take these effect into account we numerically evaluated free energy and tension for normalized effective adhesion levels presented in table 3, see figure 13. We observe that the normalized induced tension is greatly reduced when compared to situation with maximal cover. Moreover, in many cases the maximal cover even correspond to situation where the free energy would have increase when compared to a non-adhering situation, $\mathcal{F} > 0$, see dashed lines figure 13. In table 4 we render the numerical values for tension at the minimum of the free energy for the manufactured pillars with and without deposition and for hypothetical flat pillars of equal size as well. Despite the fact that the tension is greatly reduced with respect to our original estimates, the pillars with 85 nm and 100 nm induce tension one or two orders of magnitude higher than

			ρ [nm]		$\hat{\tau}_{\max} \equiv \lambda_{\max}$				
			85 nm	100 nm	0 nm	85 nm		100 nm	
ε	k	a	R	R	F	F	R	F	R
L	H	L	17.8	19.9	7.2×10^{-5}	3.9×10^{-4}	3.16×10^{-3}	5.0×10^{-4}	3.85×10^{-3}
L	L	L	21.8	24.2	2.4×10^{-4}	1.3×10^{-3}	7.69×10^{-3}	1.7×10^{-3}	9.09×10^{-3}
H	H	L	35.8	39.2	7.6×10^{-3}	0.04	0.11	0.05	0.12
H	L	H	35.7	40.7	0.03	0.14	0.11	0.17	0.17
H	L	L	39.0	42.9	0.03	0.14	0.28	0.17	0.30

Table 4: Induced membrane tension at the minimum of the free energy and minimal pillar's cap radius ρ required. By R we denote the rounded manufactured surfaces, while by F we denote hypothetical surfaces with equally large flat pillars. The pillar's cap radius ρ determines the maximal pillars size, which are still completely wetted. We omitted configurations from table 3 which would not adhere.

the pillars without deposition (0 nm). In absolute numbers, the 85 nm thick deposition induces tension in the range from 3.8 nN µm^{-1} to 185.9 nN µm^{-1}, depending on the exact conditions, while the 100 nm thick deposition induces tension from 4.5 nN µm^{-1} to 202.8 nN µm^{-1}. They also mostly outperform flat pillars of equal size, with the sole exception of the case with high bending rigidity, where the penalty induced by the bending rigidity overweights the benefit.

As we have already stated before [10, 13], the critical tension for cell membrane is in the range from 30 pN µm^{-1} to 75 pN µm^{-1}, which correspond to range from 0.06 to 0.15 for small stiffness $k_L = 500$ pN µm^{-1} and to range from 0.017 to 0.044 for large stiffness $k_H = 1690$ pN µm^{-1}. Comparing our values with these ranges, we observe that manufactured surfaces with deposited gold shall be bactericidal for highly adhesive bacteria whenever they adhere. This seems to be the case for matured adhesion both in *Pseudomonas aeruginosa* and *Staphylococcus aureus*, see section 4.2. When the distribution of the load between the cell wall and cell membrane as suggested by [13] is taken into account, the ranges changes to 0.17–0.43 and to 0.15–0.40 respectively. This would suggest that the manufactured surfaces would be bactericidal only for highly adhesive soft bacteria. However, Hwang *et al.* [13] base their estimate for the distribution of the tension between cell wall and membrane(s) on the assumption of the equal strain. While this is mostly true for in plane deformation of the envelope, the adhesion causes deformation predominately in the normal direction to the plane. In case of Gram-negative bacteria, lipoproteins [1] are responsible for binding of the outer membrane to the cell wall, and consequently are directly responsible for distribution of the normal load between individual layers. According to Braun [4], these anchors are evenly spaced 10 nm to 12 nm apart which is comparable to the gap between two neighboring pillars. This suggests that the outer membrane is largely unsupported in the gap and bears the load induced by the adhesion mostly alone, which means that the actual normalized critical tension might be closer to our initial estimate 0.06–0.15. Similar argument might be provided for Gramm-positive bacteria where lipoteichoic acid is suspected to fulfill similar role [1, 23].

In order to investigate what would be the best possible design given the dimensions of the pillars, we numerically found a radius of curvature maximizing the induced tension at the minimum of the free energy for all configurations listed in table 3. The results are summarized in table 5. We have found out that bacteria with very high bending rigidity adhere only to almost flat pillars, with the sole exception of soft highly adhesive bacteria adhering to large pillars. We further found out that weakly adhesive bacteria cannot induce tension in the bacterial envelope larger then the lowest estimate on the critical tension, this means that for weakly adhesive bacteria any reasonable surface configuration shall not be bactericidal. On the other hand, highly adhesive

ε	k	a	85 nm R [nm]	$\hat{\varepsilon}' \times 10^3$	$\hat{\tau}$	100 nm R [nm]	$\hat{\varepsilon}' \times 10^3$	$\hat{\tau}$
L	H	H	19 085.3	0.6	3.90×10^{-4}	8220.4	0.6	4.96×10^{-4}
L	H	L	123.3	0.6	9.87×10^{-3}	146.7	0.6	0.01
L	L	H	7291.0	2.0	1.30×10^{-3}	5079.1	2.0	1.65×10^{-3}
L	L	L	94.3	1.8	0.02	111.9	1.9	0.02
H	H	H	2786.0	59.0	0.04	3139.3	59.0	0.05
H	H	L	49.9	58.8	0.13	59.0	58.9	0.14
H	L	H	2785.5	200.0	0.14	55.5	96.2	0.20
H	L	L	44.3	199.2	0.29	51.8	199.4	0.32

Table 5: Radius of curvature R maximizing the induced tension $\hat{\tau}$ at the minimum of the free energy for a given pillar's cap radius ρ_{fit} (see table 1) for various combinations of adhesion, envelope stiffness and bending rigidity.

bacteria can induce large enough tension in their envelope for the surface to bactericidal, with the exception of stiff strongly adhering bacteria with envelope with large bending rigidity. The optimal radius of curvature was found to be from 44 nm to 50 nm for pillars with cap's radius $\rho = 41$ nm, and from 52 nm to 59 nm for $\rho = 46.2$ nm. This gives us an optimal radius of curvature to pillar's cap radius ratio between 1.08–1.25 for highly adhesive bacteria with low bending rigidity, while low adhesive properties shift this optimal ratio towards 2.3–3.2. Note also, that estimated parameters pillars with 130 nm thick deposition, see table 1, are closer to the optimal ratio, which can explain their higher bactericidal activity.

A Numerical solution

In order to solve numerically the constraint from (11) we first introduce re-scaled parameters

$$s_A = \frac{S_A}{d^2}, \qquad s_B = \frac{S_B}{d^2}, \qquad \hat{\varepsilon}' = \frac{\varepsilon'(R)}{k}, \qquad x = \lambda + 1$$

which simplifies the constraint to

$$\frac{s_A}{(x-\hat{\varepsilon}')^2} + \frac{s_B}{x^2} = 1.$$

This can be reformulated as a problem of finding a fixed point

$$x = f(x) = \hat{\varepsilon}' + \sqrt{s_A + s_B\left(1 - \frac{\hat{\varepsilon}'}{x}\right)^2}, \tag{14}$$

which can be solved iteratively $x_{n+1} = f(x_n)$ with initial seed $x_0 = \hat{\varepsilon}'$.

To prove that this fixed point scheme converges according to the Banach fixed point theorem, we need to determine where $f(x)$ is contraction. As we are restricted to positive real number, we will take advantage of metric

$$d(x,y) = \left|\frac{1}{x} - \frac{1}{y}\right|.$$

We need to determine when

$$d(f(x), f(y)) < d(x,y),$$

thus

$$\begin{aligned}
d(f(x),f(y)) &= \frac{\left|\sqrt{s_A + s_B\left(1-\frac{\hat{\varepsilon}'}{x}\right)^2} - \sqrt{s_A + s_B\left(1-\frac{\hat{\varepsilon}'}{y}\right)^2}\right|}{\left|\hat{\varepsilon}' + \sqrt{s_A + s_B\left(1-\frac{\hat{\varepsilon}'}{x}\right)^2}\right|\left|\hat{\varepsilon}' + \sqrt{s_A + s_B\left(1-\frac{\hat{\varepsilon}'}{y}\right)^2}\right|} \\
&\le \frac{1}{\left(\sqrt{s_A}+\hat{\varepsilon}'\right)^2}\left|\sqrt{s_A + s_B\left(1-\frac{\hat{\varepsilon}'}{x}\right)^2} - \sqrt{s_A + s_B\left(1-\frac{\hat{\varepsilon}'}{y}\right)^2}\right| \\
&= \frac{s_B}{\left(\sqrt{s_A}+\hat{\varepsilon}'\right)^2}\frac{\left|\left(1-\frac{\hat{\varepsilon}'}{x}\right)^2 - \left(1-\frac{\hat{\varepsilon}'}{y}\right)^2\right|}{\left|\sqrt{s_A + s_B\left(1-\frac{\hat{\varepsilon}'}{x}\right)^2} + \sqrt{s_A + s_B\left(1-\frac{\hat{\varepsilon}'}{y}\right)^2}\right|} \\
&\le \frac{s_B\hat{\varepsilon}'}{2\sqrt{s_A}\left(\sqrt{s_A}+\hat{\varepsilon}'\right)^2}\left|\frac{\hat{\varepsilon}'}{x^2} - \frac{\hat{\varepsilon}'}{y^2} - \frac{2}{x} + \frac{2}{y}\right| = \frac{s_B\hat{\varepsilon}'}{2\sqrt{s_A}\left(\sqrt{s_A}+\hat{\varepsilon}'\right)^2}\left|\hat{\varepsilon}'\left(\frac{1}{x}+\frac{1}{y}\right) - 2\right| d(x,y),
\end{aligned}$$

from where follow that the function is contraction when

$$\frac{s_B\hat{\varepsilon}'}{2\sqrt{s_A}\left(\sqrt{s_A}+\hat{\varepsilon}'\right)^2}\left|\hat{\varepsilon}'\left(\frac{1}{x}+\frac{1}{y}\right) - 2\right| < 1,$$

which can be further rewritten as

$$\left|\hat{\varepsilon}'\left(\frac{1}{x}+\frac{1}{y}\right) - 2\right| < \frac{2\sqrt{s_A}\left(\sqrt{s_A}+\hat{\varepsilon}'\right)^2}{s_B\hat{\varepsilon}'},$$

or

$$\frac{2}{\hat{\varepsilon}'}\left[1-\frac{\sqrt{s_A}\left(\sqrt{s_A}+\hat{\varepsilon}'\right)^2}{s_B\hat{\varepsilon}'}\right] < \frac{1}{x}+\frac{1}{y} < \frac{2}{\hat{\varepsilon}'}\left[1+\frac{\sqrt{s_A}\left(\sqrt{s_A}+\hat{\varepsilon}'\right)^2}{s_B\hat{\varepsilon}'}\right].$$

Consequently

$$\frac{\hat{\varepsilon}'}{1+\frac{\sqrt{s_A}(\sqrt{s_A}+\hat{\varepsilon}')^2}{s_B\hat{\varepsilon}'}} < x < \frac{\hat{\varepsilon}'}{1-\frac{\sqrt{s_A}(\sqrt{s_A}+\hat{\varepsilon}')^2}{s_B\hat{\varepsilon}'}},$$

where the upper bound is applicable only if $\sqrt{s_A}\left(\sqrt{s_A}+\hat{\varepsilon}'\right)^2 \le s_B\hat{\varepsilon}'$.

A.1 Maximal cover

For maximal cover of spherical caps it is valid

$$s_A = 2\pi\left(\frac{R}{d}\right)^2, \qquad s_B = 1-\pi\left(\frac{R}{d}\right)^2.$$

By inserting these values inside (14) we obtain

$$x-\hat{\varepsilon}' = \sqrt{\left(1-\frac{\hat{\varepsilon}'}{x}\right)^2+\pi\left(\frac{R}{d}\right)^2\left[2-\left(1-\frac{\hat{\varepsilon}'}{x}\right)^2\right]},$$

which can be further simplified to

$$x = \sqrt{1+\pi\left(\frac{R}{d}\right)^2\left[\frac{2}{\left(1-\frac{\hat{\varepsilon}'}{x}\right)^2}-1\right]}.$$

If we assume[1] that $\frac{R}{d}\ll 1$ and $\hat{\varepsilon}'\ll x$ the solution becomes simply

$$x \approx 1+\frac{\pi}{2}\left(\frac{R}{d}\right)^2$$

independently of the level of adhesion. Note, that by using the result the second condition can be refined to $\hat{\varepsilon}'\ll 1$. Thus in the small radius of curvature and weak effective adhesion we obtain that the tension scales as

$$\tau \propto R^2. \tag{15}$$

In the case $\hat{\varepsilon}'=1$ the situation becomes more complicated

$$x = \sqrt{1+\pi\left(\frac{R}{d}\right)^2\left[\frac{2x^2}{(x-1)^2}-1\right]}. \tag{16}$$

However if we assume that the expansion in $\frac{R}{d}$ is still valid, we approximate the equation by

$$x \approx 1+\frac{\pi}{2}\left(\frac{R}{d}\right)^2\frac{x^2+2x-1}{(x-1)^2},$$

[1]These assumptions are motivated by previous numerical results.

which can be further simplified to

$$x \approx 1 + \sqrt[3]{\frac{\pi}{2}} \left(\frac{R}{d}\right)^{\frac{2}{3}} \left(x^2 + 2x - 1\right)^{\frac{1}{3}} \approx 1 + \sqrt[3]{\pi} \left(\frac{R}{d}\right)^{\frac{2}{3}}.$$

Note, that backward substitution to (16) confirms the validity of the expansion. In this particular case tension scales with radius of curvature like

$$\tau \propto R^{\frac{2}{3}}. \tag{17}$$

A.2 Free energy minimum

In the case when the global minimum of the free energy $\mathcal{F}$ as a function of wetting angle is inside the region of $\theta \in (0, \frac{\pi}{2})$, an additional condition has to be fulfilled (10). Note, that for the purpose of this analysis the wetting angle θ is equivalent to the pillar's cap radius ρ, which allow us to rewrite the first equation in (10) as

$$\partial_\rho \mathcal{F}\, \partial_\lambda g = \partial_\lambda \mathcal{F}\, \partial_\rho g.$$

This can be further expanded to

$$-\lambda \left[\frac{1}{\sqrt{1 - \frac{\rho^2}{R^2}\left(1 - \frac{\hat{\varepsilon}'}{1+\lambda}\right)^2}} - 1 \right] = \frac{\lambda^2 - (2\lambda + 1)\,\hat{\varepsilon}' + \hat{\varepsilon}'^2}{\sqrt{1 - \frac{\rho^2}{R^2}\left(1 - \frac{\hat{\varepsilon}'}{1+\lambda}\right)^2}} - \lambda^2$$

and simplified to

$$\sqrt{1 - \frac{\rho^2}{R^2}} = \frac{1 - \frac{\hat{\varepsilon}'}{x-1}}{1 - \frac{\hat{\varepsilon}'}{x}}. \tag{18}$$

This leads to

$$s_A = 2\pi \left(\frac{R}{d}\right)^2 \frac{\hat{\varepsilon}'}{x\,(x-1)\left(1 - \frac{\hat{\varepsilon}'}{x}\right)}, \qquad s_B = 1 - 2\pi \left(\frac{R}{d}\right)^2 \frac{\hat{\varepsilon}'\left(1 - \frac{\hat{\varepsilon}}{x} - \frac{\hat{\varepsilon}}{2x(x-1)}\right)}{x\,(x-1)\left(1 - \frac{\hat{\varepsilon}'}{x}\right)^2},$$

so the (14) is simplified to

$$x - \hat{\varepsilon}' = \sqrt{\left(1 - \frac{\hat{\varepsilon}'}{x}\right)^2 + 2\pi\left(\frac{R}{d}\right)^2 \frac{\hat{\varepsilon}'}{x\,(x-1)\left(1 - \frac{\hat{\varepsilon}'}{x}\right)} \left[1 - \left(1 - \frac{\hat{\varepsilon}'}{x}\right)\left(1 - \frac{\hat{\varepsilon}'}{x} - \frac{\hat{\varepsilon}'}{2x(x-1)}\right)\right]},$$

which can be further simplified

$$x = \sqrt{1 + 2\pi \left(\frac{R}{d}\right)^2 \frac{\hat{\varepsilon}'}{x\,(x-1)\left(1 - \frac{\hat{\varepsilon}'}{x}\right)^2} \left[\frac{1}{1 - \frac{\hat{\varepsilon}'}{x}} - \left(1 - \frac{\hat{\varepsilon}'}{x} - \frac{\hat{\varepsilon}'}{2x(x-1)}\right)\right]}.$$

Following the same logic as in the previous section we obtain

$$x^2 - 1 = 2\pi \left(\frac{R}{d}\right)^2 \frac{\hat{\varepsilon}'^2}{2x^2\,(x-1)^2\left(1 - \frac{\hat{\varepsilon}'}{x}\right)^3} \left[4x - 3 - \frac{\hat{\varepsilon}'}{x}\,(2x - 1)\right],$$

which we rewrite to

$$(x-1)^3 = \pi \left(\frac{R}{d}\right)^2 \frac{\hat{\varepsilon}'^2}{x^2(1+x)\left(1-\frac{\hat{\varepsilon}'}{x}\right)^3} \left[4x - 3 - \frac{\hat{\varepsilon}'}{x}(2x-1)\right],$$

and, under the assumptions $1 \gg x - 1 \gg \hat{\varepsilon}' > 0$, approximate by

$$(x-1)^3 \approx \frac{\pi}{2}\left(\frac{R}{d}\right)^2 \hat{\varepsilon}'^2$$

or simply as

$$x \approx 1 + \sqrt[3]{\frac{\pi}{2}}\hat{\varepsilon}'^{\frac{2}{3}}\left(\frac{R}{d}\right)^{\frac{2}{3}}, \qquad \tau \propto \hat{\varepsilon}'^{\frac{2}{3}}\left(\frac{R}{d}\right)^{\frac{2}{3}}. \tag{19}$$

Inserting this result back to the equation for the radius (18) we obtain an estimate for the wetting angle θ as

$$\cos\theta = \sqrt{1 - \frac{\rho^2}{R^2}} \approx 1 - \sqrt[3]{\frac{2}{\pi}}\hat{\varepsilon}'^{\frac{1}{3}}\left(\frac{R}{d}\right)^{-\frac{2}{3}}. \tag{20}$$

As the correction term is not necessarily small at all cases, further expansion is possible only for small adhesion energy and large curvature radius

$$\theta \approx \sqrt[6]{\frac{16}{\pi}}\hat{\varepsilon}'^{\frac{1}{6}}\left(\frac{R}{d}\right)^{-\frac{1}{3}}.$$

In this regime we can also estimate the maximal tension as a tension at $\frac{\rho}{d} = \frac{1}{2}$. First, we determine the radius of curvature $R_{\max}$ at the maximal tension as a function of normalized effective adhesion $\hat{\varepsilon}'$ from (20),

$$\frac{R_{\max}}{d} \approx \sqrt[4]{\frac{\pi}{2^{10}\hat{\varepsilon}'}}.$$

Then from (19) we obtain the maximal tension as a function of normalized effective adhesion

$$\hat{\tau}_{\max} \approx \frac{1}{4}\sqrt{\pi\hat{\varepsilon}'},$$

or alternatively as a function of the radius of curvature at maximal tension

$$\hat{\tau}_{\max} \approx \frac{\pi}{128}\left(\frac{R_{\max}}{d}\right)^{-2}.$$

A.3 Flat pillars

Last case is maximally wetted flat pillars. In this case

$$s_A = \frac{\pi}{4}, \qquad s_B = 1 - \frac{\pi}{4}.$$

Inserting these relations to (14) we obtain

$$x - \hat{\varepsilon}' = \sqrt{1 - \frac{\hat{\varepsilon}'}{x}\left(1 - \frac{\pi}{4}\right)\left(2 - \frac{\hat{\varepsilon}'}{x}\right)}$$

which in case of small normalized effective adhesion $\hat{\varepsilon}' \ll 1$ can be further approximated by

$$x \approx 1 + \frac{\pi}{4}\hat{\varepsilon}'.$$

Consequently

$$\hat{\tau} \approx \frac{\pi}{4}\hat{\varepsilon}'.$$

In the particular case when $\hat{\varepsilon}' = 1$ we obtain

$$x - 1 = \sqrt{1 - \frac{1}{x}\left(1 - \frac{\pi}{4}\right)\left(2 - \frac{1}{x}\right)}.$$

By using the numerical computation as the ansatz we perturb the equation around $x = 2$ and obtain in the linear order

$$x \approx \frac{5\pi + 44}{\pi + 28}, \qquad \hat{\tau} \approx \frac{4\pi + 16}{\pi + 28}.$$

References

[1] George K. Auer and Douglas B. Weibel. Bacterial Cell Mechanics. *Biochemistry*, 56(29): 3710–3724, jul 2017. ISSN 0006-2960. doi: 10.1021/acs.biochem.7b00346. URL http://pubs.acs.org/doi/10.1021/acs.biochem.7b00346.

[2] H. Bermúdez, D. A. Hammer, and D. E. Discher. Effect of Bilayer Thickness on Membrane Bending Rigidity. *Langmuir*, 20(3):540–543, feb 2004. ISSN 0743-7463. doi: 10.1021/la035497f. URL https://pubs.acs.org/doi/10.1021/la035497f.

[3] David Boal and David H Boal. *Mechanics of the Cell*. Cambridge University Press, 2012.

[4] Volkmar Braun. Molecular organization of the rigid layer and the cell wall of escherichia coli. *The Journal of Infectious Diseases*, 128:S9–S16, 1973. ISSN 00221899. doi: 10.2307/30106028. URL http://www.jstor.org/stable/30106028.

[5] J. P. Busalmen and S. R. de Sanchez. Adhesion of Pseudomonas fluorescens (ATCC 17552) to Nonpolarized and Polarized Thin Films of Gold. *Applied and Environmental Microbiology*, 67(7):3188–3194, jul 2001. ISSN 0099-2240. doi: 10.1128/AEM.67.7.3188-3194.2001. URL http://aem.asm.org/cgi/doi/10.1128/AEM.67.7.3188-3194.2001.

[6] Vera Carniello, Brandon W. Peterson, Henny C. van der Mei, and Henk J. Busscher. Physicochemistry from initial bacterial adhesion to surface-programmed biofilm growth. *Advances in Colloid and Interface Science*, 261:1–14, nov 2018. ISSN 00018686. doi: 10.1016/j.cis.2018.10.005. URL https://linkinghub.elsevier.com/retrieve/pii/S000186861830229X.

[7] Kenneth K. Chung, James F. Schumacher, Edith M. Sampson, Robert A. Burne, Patrick J. Antonelli, and Anthony B. Brennan. Impact of engineered surface microtopography on biofilm formation of Staphylococcus aureus. *Biointerphases*, 2(2):89–94, jun 2007. ISSN 1934-8630. doi: 10.1116/1.2751405. URL http://avs.scitation.org/doi/10.1116/1.2751405.

[8] Kerem Ege, N.B. Roozen, Quentin Leclère, and Renaud G. Rinaldi. Assessment of the apparent bending stiffness and damping of multilayer plates; modelling and experiment. *Journal of Sound and Vibration*, 426:129–149, jul 2018. ISSN 0022460X. doi: 10.1016/j.jsv.2018.04.013. URL https://linkinghub.elsevier.com/retrieve/pii/S0022460X18302384.

[9] Dmitry A. Fedosov, Bruce Caswell, and George Em Karniadakis. Systematic coarse-graining of spectrin-level red blood cell models. *Computer Methods in Applied Mechanics and Engineering*, 199(29-32):1937–1948, jun 2010. ISSN 00457825. doi: 10.1016/j.cma.2010.02.001. URL http://linkinghub.elsevier.com/retrieve/pii/S0045782510000599.

[10] R.D. Groot and K.L. Rabone. Mesoscopic Simulation of Cell Membrane Damage, Morphology Change and Rupture by Nonionic Surfactants. *Biophysical Journal*, 81(2):725–736, aug 2001. ISSN 00063495. doi: 10.1016/S0006-3495(01)75737-2. URL https://linkinghub.elsevier.com/retrieve/pii/S0006349501757372.

[11] W Helfrich. Elastic Properties of Lipid Bilayers: Theory and Possible Experiments. *Zeitschrift für Naturforschung C*, 28(11-12):044105, jan 1973. ISSN 1865-7125. doi: 10.1515/znc-1973-11-1209. URL https://www.degruyter.com/view/j/znc.1973.28.issue-11-12/znc-1973-11-1209/znc-1973-11-1209.xml.

[12] Qiaoyun Huang, Huayong Wu, Peng Cai, Jeremy B. Fein, and Wenli Chen. Atomic force microscopy measurements of bacterial adhesion and biofilm formation onto clay-sized particles. *Scientific Reports*, 5(1):16857, dec 2015. ISSN 2045-2322. doi: 10.1038/srep16857. URL http://dx.doi.org/10.1038/srep16857 http://www.nature.com/articles/srep16857 .

[13] Hyea Hwang, Nicolò Paracini, Jerry M. Parks, Jeremy H. Lakey, and James C. Gumbart. Distribution of mechanical stress in the Escherichia coli cell envelope. *Biochimica et Biophysica Acta (BBA) - Biomembranes*, 1860(12):2566–2575, dec 2018. ISSN 00052736. doi: 10.1016/j.bbamem.2018.09.020. URL https://linkinghub.elsevier.com/retrieve/pii/S0005273618302931.

[14] K. L. Johnson. Adhesion and friction between a smooth elastic spherical asperity and a plane surface. *Proceedings of the Royal Society A: Mathematical, Physical and Engineering Sciences*, 453(1956):163–179, 1997. ISSN 1364-5021. doi: 10.1098/rspa.1997.0010. URL http://rspa.royalsocietypublishing.org/cgi/doi/10.1098/rspa.1997.0010.

[15] K.L. Johnson and J.A. Greenwood. An Adhesion Map for the Contact of Elastic Spheres. *Journal of Colloid and Interface Science*, 192(2):326–333, aug 1997. ISSN 00219797. doi: 10.1006/jcis.1997.4984. URL http://198.81.200.2/science/article/B6WHR-45KV0OY-44/2/6260888b90eaddeacf6345ca1f2d9b3b http://linkinghub.elsevier.com/retrieve/pii/S0021979797949845 .

[16] M. G. Katsikogianni and Y. F. Missirlis. Bacterial adhesion onto materials with specific surface chemistries under flow conditions. *Journal of Materials Science: Materials in Medicine*, 21(3):963–968, mar 2010. ISSN 0957-4530. doi: 10.1007/s10856-009-3975-y. URL http://link.springer.com/10.1007/s10856-009-3975-y.

[17] M.G. Katsikogianni and Y.F. Missirlis. Interactions of bacteria with specific biomaterial surface chemistries under flow conditions. *Acta Biomaterialia*, 6(3):1107–1118, mar 2010. ISSN 17427061. doi: 10.1016/j.actbio.2009.08.006. URL https://linkinghub.elsevier.com/retrieve/pii/S1742706109003419.

[18] Jiuyi Li, Henk J. Busscher, Jan J. T. M. Swartjes, Yun Chen, Akshay K. Harapanahalli, Willem Norde, Henny C. van der Mei, and Jelmer Sjollema. Residence-time dependent cell wall deformation of different Staphylococcus aureus strains on gold measured using surface-enhanced-fluorescence. *Soft Matter*, 10(38):7638–7646, 2014. ISSN 1744-683X. doi: 10.1039/C4SM00584H. URL http://xlink.rsc.org/?DOI=C4SM00584H.

[19] Natasa Mitik-Dineva, James Wang, Vi Khanh Truong, Paul Stoddart, Francois Malherbe, Russell J. Crawford, and Elena P. Ivanova. Escherichia coli, pseudomonas aeruginosa, and staphylococcus aureus attachment patterns on glass surfaces with nanoscale roughness. *Current Microbiology*, 58(3):268–273, Mar 2009. ISSN 1432-0991. doi: 10.1007/s00284-008-9320-8. URL `https://doi.org/10.1007/s00284-008-9320-8`.

[20] Nobuyuki Otsu. A Threshold Selection Method from Gray-Level Histograms. *IEEE Transactions on Systems, Man, and Cybernetics*, 9(1):62–66, jan 1979. ISSN 0018-9472. doi: 10.1109/TSMC.1979.4310076. URL `http://ieeexplore.ieee.org/document/4310076/`.

[21] Rob Phillips, Tristan Ursell, Paul Wiggins, and Pierre Sens. Emerging roles for lipids in shaping membrane-protein function. *Nature*, 459(7245):379–385, may 2009. ISSN 0028-0836. doi: 10.1038/nature08147. URL `http://www.nature.com/articles/nature08147`.

[22] Sergey Pogodin, Jafar Hasan, Vladimir A. Baulin, Hayden K. Webb, Vi Khanh Truong, The Hong Phong Nguyen, Veselin Boshkovikj, Christopher J. Fluke, Gregory S. Watson, Jolanta A. Watson, Russell J. Crawford, and Elena P. Ivanova. Biophysical Model of Bacterial Cell Interactions with Nanopatterned Cicada Wing Surfaces. *Biophysical Journal*, 104(4):835–840, feb 2013. ISSN 00063495. doi: 10.1016/j.bpj.2012.12.046. URL `http://dx.doi.org/10.1016/j.bpj.2012.12.046` `http://linkinghub.elsevier.com/retrieve/pii/S0006349513000039` .

[23] Nathalie T. Reichmann, Carolina Piçarra Cassona, João M. Monteiro, Amy L. Bottomley, Rebecca M. Corrigan, Simon J. Foster, Mariana G. Pinho, and Angelika Gründling. Differential localization of LTA synthesis proteins and their interaction with the cell division machinery in Staphylococcus aureus. *Molecular Microbiology*, 92(2):273–286, apr 2014. ISSN 0950382X. doi: 10.1111/mmi.12551. URL `http://doi.wiley.com/10.1111/mmi.12551`.

[24] Bart Smeets, Maxim Cuvelier, Jiri Pešek, and Herman Ramon. The Effect of Cortical Elasticity and Active Tension on Cell Adhesion Mechanics. *Biophysical Journal*, 116(5):930–937, mar 2019. ISSN 00063495. doi: 10.1016/j.bpj.2019.01.015. URL `https://linkinghub.elsevier.com/retrieve/pii/S0006349519300505`.
